# Excessive immunosuppression by regulatory T cells antagonizes T cell response to schistosome infection in PD-1-deficient mice

**DOI:** 10.1371/journal.ppat.1010596

**Published:** 2022-06-06

**Authors:** Liaoxun Lu, Tianhan Li, Xinyu Feng, Zhilong Liu, Yang Liu, Tianzhu Chao, Yanrong Gu, Rong Huang, Fanghui Zhang, Le He, Binhui Zhou, Eryan Kong, Zhuangzhuang Liu, Xugang Wang, Zhijun Chen, Hui Wang, Marie Malissen, Bernard Malissen, Lichen Zhang, Yinming Liang

**Affiliations:** 1 Laboratory of Genetic Regulators in the Immune System, Henan Collaborative Innovation Center of Molecular Diagnosis and Laboratory Medicine, School of Laboratory Medicine, Xinxiang Medical University, Xinxiang, China; 2 Henan Key Laboratory of Immunology and Targeted Therapy, Xinxiang Medical University, Xinxiang, China; 3 Institute of Psychiatry and Neuroscience, Xinxiang Medical University, Xinxiang, China; 4 School of Public Health, Xinxiang Medical University, Xinxiang, China; 5 The First Affiliated Hospital of Xinxiang Medical University, Xinxiang, China; 6 Centre d’Immunologie de Marseille-Luminy, Aix Marseille Université, Marseille, France; University of California Riverside, UNITED STATES

## Abstract

Schistosomiasis is caused by parasitic flatworms known as schistosomes and affects over 200 million people worldwide. Prevention of T cell exhaustion by blockade of PD-1 results in clinical benefits to cancer patients and clearance of viral infections, however it remains largely unknown whether loss of PD-1 could prevent or cure schistosomiasis in susceptible mice. In this study, we found that *S*. *japonicum* infection dramatically induced PD-1 expression in T cells of the liver where the parasites chronically inhabit and elicit deadly inflammation. Even in mice infected by non-egg-producing unisex parasites, we still observed potent induction of PD-1 in liver T cells of C57BL/6 mice following *S*. *japonicum* infection. To determine the function of PD-1 in schistosomiasis, we generated PD-1-deficient mice by CRISPR/Cas9 and found that loss of PD-1 markedly increased T cell count in the liver and spleen of infected mice. IL-4 secreting Th2 cells were significantly decreased in the infected PD-1-deficient mice whereas IFN-γ secreting CD4^+^ and CD8^+^ T cells were markedly increased. Surprisingly, such beneficial changes of T cell response did not result in eradication of parasites or in lowering the pathogen burden. In further experiments, we found that loss of PD-1 resulted in both beneficial T cell responses and amplification of regulatory T cells that prevented PD-1-deficient T cells from unleashing anti-parasite activity. Moreover, such PD-1-deficient Tregs exert excessive immunosuppression and express larger amounts of adenosine receptors CD39 and CD73 that are crucial for Treg-mediated immunosuppression. Our experimental results have elucidated the function of PD-1 in schistosomiasis and provide novel insights into prevention and treatment of schistosomiasis on the basis of modulating host adaptive immunity.

## Author summary

Chronic schistosome infection leads to exaggerated upregulation of PD-1 in the liver, and loss of PD-1 markedly increased T cell presence in the liver of schistosome infected mice, which was accompanied by suppressed Th2 cytokines but markedly increased IFN-γ secretion in CD4^+^ and CD8^+^ T cells. The beneficial T cell response did not result in eradication of parasites or lowering the pathogen burden. Loss of PD-1 also resulted in amplification of Tregs and excessive Treg-mediated immunosuppression may prevent T cells from unleashing anti-parasitic immunity.

## Introduction

PD-1 blockade antibodies have become the front-line treatment for cancer by modulating T cell responses, even though their efficacy varies among patients and different types of cancers [1–3]. However, in comparison to cancer studies, knowledge of the role of PD-1 in chronic schistosome infection is very limited. Moreover, the mechanisms underlying PD-1 functions in various disease settings are still incompletely elucidated. Therefore, investigations into the function of PD-1 in different infection models are necessary to obtain a more complete and precise understanding of this important immune checkpoint molecule. More importantly, in both cancer studies and infectious disease studies, blockade of PD-1 has been shown to result in conflicting results, which suggests that modulation of T cell responses by PD-1 can be context dependent.

Schistosomiasis affects over 200 million people worldwide, and hyporesponsive T cells are implicated in chronic infection [4–6]. T cells are essential for host immunity against tumors and pathogens, and PD-1 blockade is believed to be promising for clearance of chronic infection and tumor eradication [7,8], however its roles in containing pathogens including schistosome parasites are far less characterized. Our previous work using genetically modified SD rats deficient in T cells confirmed that natural resistance to schistosome infection in rats was dependent on T cells [9], but the modulation of T cell responsiveness by using PD-1-deficient animals during chronic infection of *S*. *japonicum* has not been previously performed. The benefits of inhibiting PD-1 in melanoma and several other types of cancer are evident, and in viral infections loss of PD-1 significantly reduces viral load in animal models [10,11]. It is important to note that anti-PD-1 treatment may not always result in therapeutic effects. In a primate model, blocking PD-1 was shown to cause more severe pathology in rhesus macaques infected with *Mycobacterium tuberculosis* [12]. In addition, recent studies also revealed that loss of PD-1 expressed by Tregs resulted in increased Treg cell differentiation, and PD-1 deficiency ameliorated autoimmunity in NOD mice [13]. Such results are in line with findings from cancer immunotherapies in which PD-1 blockade results in increased Treg suppression [14], which needs further verification as some studies reported opposite results that PD-1 blockade could help overcome Treg suppression [15]. Surprisingly another recent study showed that decreased antigen specific T cell response in *Toxoplasma gondii* infected mice which had germline deletion of PD-1 or Treg specific ablation of PD-1, was associated with increased parasite burden [16]. In a previous study, PD-1 blockade in mice were found to exacerbate liver pathology and enhance Th2 differentiation [17], however the consequence of PD-1 inhibition for pathogens or the T cell response in the liver were not elucidated.

In summary, even though it is well documented that PD-1 blockade results in clinic benefits to cancer patients and clearance of viral infections, the exact consequence of PD-1 blockade may vary among different diseases. Therefore, it is important to determine whether genetic deficiency of PD-1 in an animal model could ameliorate or cure schistosome infection on the susceptible C57BL/6 background. In our experiments, PD-1 upregulation following *S*. *japonicum* infection occurred obviously in T cells of the liver, where the adult parasites inhabit and parasitic eggs are trapped. In PD-1-deficient mice with 8 weeks of *S*. *japonicum* infection, we observed significantly increased numbers of T cells in the liver and they secreted significantly larger amount of IFN-γ in both CD4^+^ and CD8^+^ T cells compared to controls. Such results were opposite to the dropped IFN-γ production observed in conventional CD4^+^ T cells in inflamed pancreatic tissue in NOD mice deprived of PD-1 in Tregs [13]. Unexpectedly, such increased T cell numbers and cytokine secretion did not result in a significant reduction of pathogen burden in PD-1-deficient mice. Our experiments also found that loss of PD-1 dramatically increased the Treg frequencies and Foxp3 expression in Tregs, which were accompanied by excessive immunosuppression and larger amounts of inhibitory molecules on the surface of Tregs, including CD39 and CD73. Our experimental data confirmed the contrasting roles of PD-1deficiency during schistosome infection, by increasing T cell numbers in infected liver and IFN-γ production in T cells, but on the other hand promoting differentiation of Tregs that exert excessive immunosuppression. Such results shed light on the complexity of T cell responses against schistosome parasites and provide evidence to restore T cell activity by PD-1 blockade and inhibiting Treg differentiation for schistosomiasis treatment.

## Results

### *S*. *japonicum* infection potently induces PD-1 expression in liver T cells and inhibits T cell proliferation

**Fig 1:**
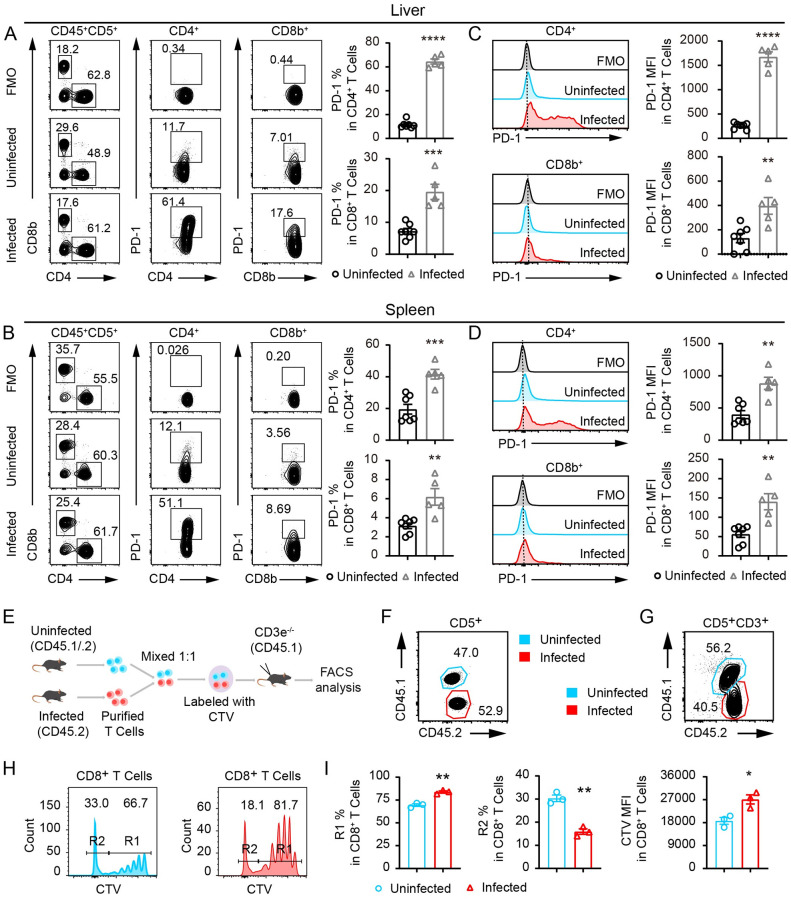
PD-1 expression induced in CD4^+^ and CD8^+^ T cells by *S*. *japonicum* infection. (A) Flow cytometric analyses of single cell suspension of the liver obtained by mechanic and enzymatic tissue dissociation with surface antigen labeling of CD45, CD5, CD4, CD8b and PD-1 from both uninfected control mice and mice infected with 30 *S*. *japonicum* cercariae for 8 weeks. Frequencies of PD-1 expressing cells were compared between two group of mice by gating the PD-1 positive compartment involving Fluorescence-minus-one or FMO control (Infected mice, n = 5; Uninfected mice, n = 7). (B) Flow cytometric analyses of single cell suspension of splenocytes obtained by mechanic and enzymatic tissue dissociation, and cells were labeled and analyzed in the same manner as shown in (A), (Infected mice, n = 5; Uninfected mice, n = 7). (C) Histogram comparison of PD-1 expression in liver CD4^+^ and CD8^+^ T cells and statistic comparisons of mean fluorescence intensity or MFI between the two group of mice analyzed in (A). (D) Histogram comparison of PD-1 expression in splenic CD4^+^ and CD8^+^ T cells and statistic comparisons of MFI between the two group of mice analyzed in (B). (E) Schematic presentation of adoptive transfer of T cells from *S*. *japonicum* infected and uninfected mice into *CD3e*^−/−^ T cell deficient recipient mice to analyze *in vivo* proliferation by CTV labeling. (F) CD45.2^+^ T cells from *S*. *japonicum* infected mice and CD45.1^+^CD45.2^+^ T cells from uninfected controls were mixed in a 1:1 ratio before transfer in T cell deficient recipient mice. (G-I) 5 days after transfer into the *CD3e*^−/−^ T cell deficient recipient mice, CD45.2^+^ T cells from *S*. *japonicum* infected mice and CD45.1^+^CD45.2^+^ T cells from uninfected controls were analyzed by flow cytometry for their frequencies (G) and levels of T cell division measured by CTV dilution including most divided cells in R2 gating and less divided cells in R1 gating (H), which were further compared by MFI and percentages of R2 and R1 (I). Data represent the mean ± s.e.m. Statistical significance was assessed by unpaired Student’s *t*-test or non-parametric unpaired Mann-Whitney test and indicated by * *P*<0.05, ** *P*<0.01, *** *P*<0.001, **** *P*<0.0001.

To analyze T cell responses and PD-1-mediated exhaustion in schistosomiasis, we infected C57BL/6 wildtype (WT) mice with 30 cercariae of *S*. *japonicum* as described previously [9]. As schistosomiasis is an infectious disease with devastating liver pathology, we analyzed T cell responses in infected murine livers. By dissociating the liver tissue with programmed mechanic force and enzymes as described in the Material and Methods section, we analyzed the phenotype of T cells in the liver of infected mice and the uninfected controls. In the livers of *S*. *japonicum* infected mice, expression of PD-1 in both CD4^+^ and CD8^+^ T cells was potently induced (Fig 1A), even more so than that observed in splenocytes (Fig 1B). In particular, 8 weeks after infection, the average frequency of PD-1 expressing cells peaked at 64.5% in CD4^+^ T cells, which was a 5.6-fold increase from the uninfected controls. We also compared the mean fluorescence intensity (MFI) of PD-1 in both CD4^+^ and CD8^+^ T cells in the liver and spleen of infected mice to the uninfected controls. PD-1 levels were significantly higher in infected mice for both CD4^+^ and CD8^+^ T cells in the liver (Fig 1C) and the spleen (Fig 1D). Next, we analyzed PD-1 expression in mice subjected to shorter term *S*. *japonicum* infection. Strikingly, 4 weeks after infection, the PD-1 expression was already potently induced in both CD4^+^ and CD8^+^ T cells in the liver and spleen (S1A and S1B Fig). We found that within 4 weeks of *S*. *japonicum* infection, liver CD4^+^ T cells expressed exaggerated amounts of PD-1, and 50.6% of CD4^+^ T cells in the liver were positive for PD-1 while 22.1% of CD4^+^ T cells in spleen expressed PD-1 in infected mice. In the same mice infected for 4 weeks, 17.3% of CD8^+^ T cells in the liver were positive for PD-1 while 5.1% of CD8^+^ T cells in the spleen expressed PD-1(S1A and S1B Fig). With these data, we clearly show that it is necessary to analyze T cell responses in the liver where the parasitic eggs are trapped and pathology develops.

We further sought to elucidate whether such potent induction of PD-1 expression in T cells was dependent on egg antigens by infecting the mice with unisex worms which were not able to produce parasitic eggs or form egg granulomas. Thirty unisex cercariae of *S*. *japonicum* released from a single snail were used to infect each B6 mouse for 8 weeks, and we found that in the egg/granuloma-free livers of the infected animals (S2A Fig), PD-1 was still significantly induced by parasitic worms in CD4^+^ T cells with an increase from 12.4% of PD-1^+^ cells in uninfected controls to 33.8% in the infected mice, but such an increase was not observed in CD8^+^ T cells (S2B and S2C Fig). Such data indicates that PD-1-mediated T cell exhaustion can be caused by both parasitic worms and eggs, with the worms preferentially affecting PD-1 expression in CD4^+^ T cells.

To determine proliferative capability of T cells from *S*. *japonicum* infected mice, we co-transferred peripheral T cells from infected mice and uninfected controls into the T cell deficient CD45.1^+^ *CD3e*^*−/−*^ recipient mice, in which the transferred T cells were tested for homeostatic proliferation *in vivo*. In the transfer experiments, peripheral T cells from the two groups of donors were mixed in a 1:1 ratio, with CD45.2^+^ T cells from *S*. *japonicum* infected mice and CD45.1^+^CD45.2^+^ T cells from uninfected controls (Fig 1E and 1F). Five days after adoptive transfer, we observed that the CD45.2^+^CD8^+^ T cells from *S*. *japonicum* infected mice were significantly less competent in proliferation *in vivo* than their uninfected CD45.1^+^CD45.2^+^ controls (Fig 1G, 1H and 1I). Our results confirmed that *S*. *japonicum* infection potently induced PD-1 expression in T cells of the liver and spleen, which was accompanied by an impaired capacity to proliferate *in vivo*.

### PD-1 deficiency significantly increases T cell numbers in the liver during chronic infection of *S*. *japonicum*

**Fig 2:**
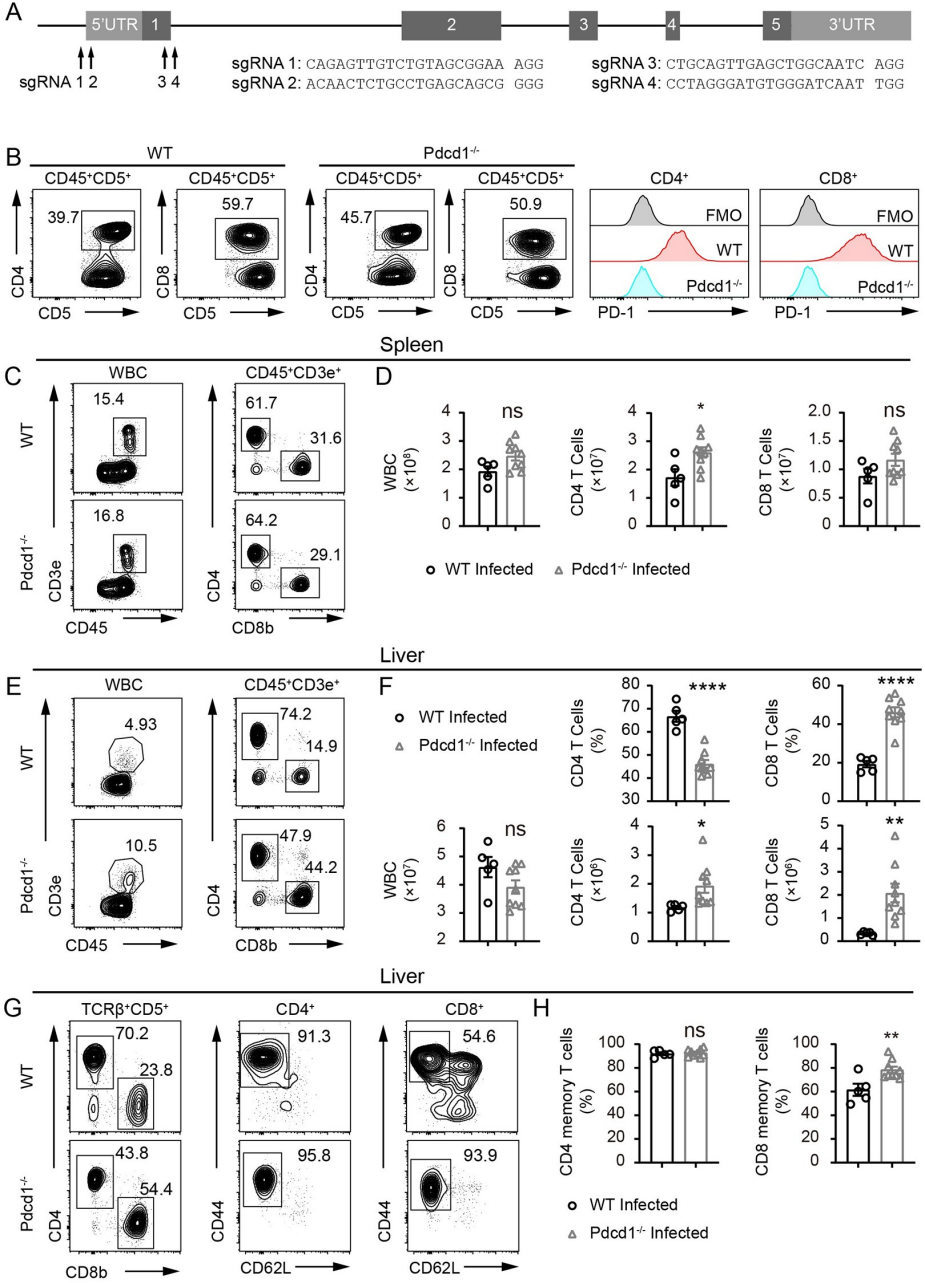
Increase of T cell presence in the liver of PD-1-deficient mice infected with *S*. *japonicum* for 8 weeks. (A) sgRNA design to target *Pdcd1* gene in C57BL/6 mice using CRISPR/Cas9 genome editing tool. (B) Flow cytometric analyses of PD-1 expression in T cells from peripheral blood of both WT and PD-1-deficient mice following 72 h treatment with anti-CD3 (3 μg/mL) and anti-CD28 (1 μg/mL) antibodies. (C-D) Flow cytometric analyses of WBCs, and T cells including CD4^+^ and CD8^+^ T cells in the spleen of WT and PD-1-deficient *Pdcd1*^*−/−*^ mice infected with *S*. *japonicum* for 8 weeks (WT mice, n = 5; KO mice, n = 9). (E-F) Flow cytometric analyses of WBCs, and T cells including CD4^+^ and CD8^+^ T cells in the livers of WT and PD-1-deficient *Pdcd1*^*−/−*^ mice infected with *S*. *japonicum* for 8 weeks (WT mice, n = 5; KO mice, n = 9). (G-H) Flow cytometric analyses of memory T cells in CD4^+^ and CD8^+^ T cell subsets in the livers of WT and PD-1-deficient mice infected with *S*. *japonicum* for 8 weeks (WT mice, n = 5; KO mice, n = 9). Data represent the mean ± s.e.m. Statistical significance was assessed by unpaired Student’s *t*-test or non-parametric unpaired Mann-Whitney test and indicated by * *P*<0.05, ** *P*<0.01, **** *P*<0.0001, ns, non-significant.

Since *S*. *japonicum* infection induces high levels of PD-1 expression in CD4^+^ and CD8^+^ T cells and impairs T cell proliferation, we aimed to generate PD-1-deficient mice and analyze the impact of PD-1 deficiency on schistosomiasis. PD-1-deficient C57BL/6 mice were established using CRISPR/Cas9 targeting of exon 1 and 5’ UTR region of *Pdcd1* gene with 4 independent sgRNAs (Fig 2A). The knockout mice were phenotypically validated by monoclonal antibody staining of PD-1 after stimulating CD4^+^ and CD8^+^ T cells with coated anti-CD3 and soluble anti-CD28 antibodies to induce PD-1 surface expression (Fig 2B). In the spleens of infected *Pdcd1*^*−/−*^ B6 mice, the CD45^+^ cells were not significantly different from infected WT controls, and the CD8^+^ T cells were also not changed in numbers. However, the CD4^+^ T cells in the spleen of PD-1-deficient mice were significantly higher in numbers when they were compared to WT controls 8 weeks after *S*. *japonicum* infection (Fig 2C and 2D). In the livers, the CD45^+^ cells of knockout and control mice were comparable in numbers 8 weeks after *S*. *japonicum* infection, however T cells, including both CD4^+^ and CD8^+^ subsets, were significantly increased in absolute numbers in knockout mice (Fig 2E and 2F). Such cell number change in the livers of PD-1-deficient mice was more dramatic for CD8^+^ T cells with 4.6-fold increase, and the CD4^+^ T cells displayed 1.6-fold increase which was associated with the drop of CD4^+^ T cell frequencies (Fig 2F). We further analyzed the memory T cells in the livers of infected mice. While the frequencies of CD44^high^ CD62L^low^ effector memory cells in CD4^+^ T cells were not significantly different, the frequencies of effector memory CD8^+^ T cells, gated on CD44^high^ CD62L^low^ compartment, were significantly higher in PD-1-deficient *Pdcd1*^*−/−*^ B6 mice (Fig 2G and 2H). However, the frequency increase in CD8^+^ memory T cells observed in the liver of infected PD-1-deficient mice was not found in the spleen. In steady state, the total T cell counts in the spleen and the liver were not significantly different in PD-1-deficient. In spleen and liver CD8^+^ effector memory frequencies were significantly higher but the CD4^+^ effector memory was not changed by PD-1 deficiency in the liver in steady state (S3A–S3D Fig). Our experiments using infected PD-1-deficient *Pdcd1*^*−/−*^ mice and WT control mice confirmed that loss of PD-1 significantly promoted T cell presence in the livers of C57BL/6 mice following *S*. *japonicum* infection. The dramatic increase of PD-1-deficient CD8^+^ T cells in the livers of infected mice indicates that PD-1 is a critical molecule modulating T cell responses during chronic *S*. *japonicum* infection.

### PD-1 deficiency suppresses Th2 differentiation and promotes IFN-γ production in T cells during chronic *S*. *japonicum* infection

**Fig 3:**
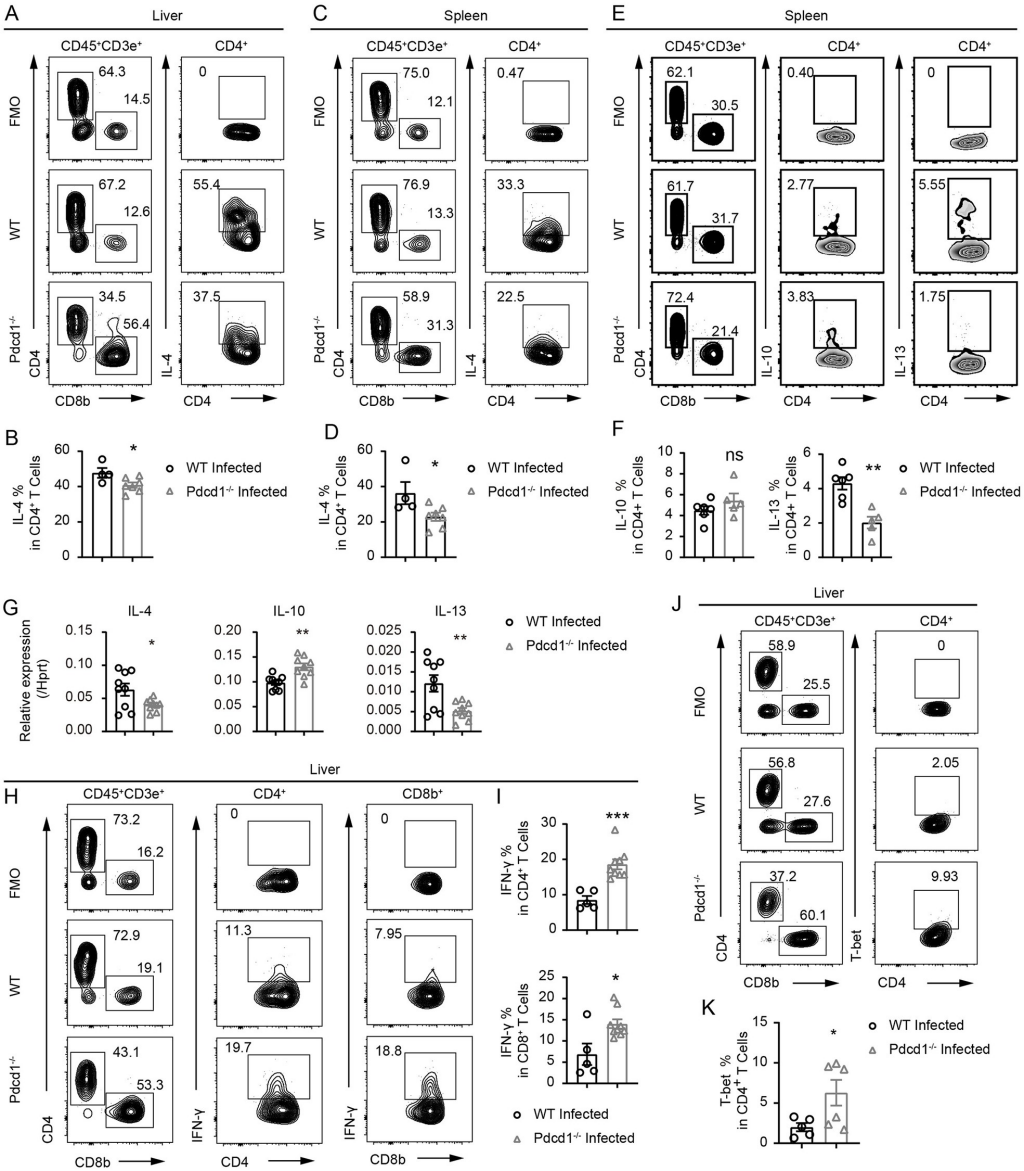
PD-1 deficiency suppresses Th2 differentiation and promotes IFN-γ production in liver T cells of the *S*. *japonicum* infected mice. (A-B) Flow cytometric analyses of IL-4 expression in T cells in the livers of WT and PD-1-deficient *Pdcd1*^*−/−*^ mice infected with *S*. *japonicum* for 8 weeks (WT mice, n = 4; KO mice, n = 7). (C-D) Flow cytometric analyses of IL-4 expression in T cells in the spleens of WT and PD-1-deficient *Pdcd1*^*−/−*^ mice infected with *S*. *japonicum* for 8 weeks (WT mice, n = 4; KO mice, n = 7). (E-F) Flow cytometric analyses of IL-10 and IL-13 expression in splenic T cells of WT and PD-1-deficient *Pdcd1*^*−/−*^ mice infected with *S*. *japonicum* for 8 weeks (WT mice, n = 6; KO mice, n = 5). (G) qRT-PCR analyses of IL-4, IL-10 and IL-13 mRNAs in T cells purified by streptavidin-coated beads from splenocytes of WT and PD-1-deficient *Pdcd1*^*−/−*^ mice infected with *S*. *japonicum* for 8 weeks (WT mice, n = 3; KO mice, n = 3; each sample analyzed in 3 replicates). (H-I) Flow cytometric analyses of IFN-γ expression in T cells in the livers of WT and PD-1-deficient mice infected with *S*. *japonicum* for 8 weeks (WT mice, n = 5; KO mice, n = 9). (J-K) Flow cytometric analyses of T-bet expression in CD4^+^ T cells in the livers of WT and PD-1-deficient mice infected with *S*. *japonicum* for 8 weeks (WT mice, n = 5; KO mice, n = 6). Data represent the mean ± s.e.m. Statistical significance was assessed by unpaired Student’s *t*-test or non-parametric unpaired Mann-Whitney test and indicated by * *P*<0.05, ** *P*<0.01, *** *P*<0.001, ns, non-significant.

After we found a significant increase in the number of T cells in infected PD-1-deficient mice compared to controls, we further analyzed the cytokine profile of T cells in infected livers and spleens. In our study, PD-1 deficiency potently increased T cell counts in the livers and spleens of infected animals, therefore we analyzed whether PD-1 deficiency could change the number of Th2 cells which are dominantly induced during schistosomiasis. Surprisingly, we did not observe increased differentiation of Th2 cells in PD-1-deficient mice 8 weeks following *S*. *japonicum* infection. Instead, in the livers of infected PD-1-deficient mice, we observed a significant reduction in IL-4 by intracellular antibody staining as compared to the infected WT controls (Fig 3A and 3B). In the spleens, we also found the same reduction of IL-4 at the protein level in infected PD-1-deficient mice (Fig 3C and 3D). In addition, we performed intracellular staining of IL-13 and observed a significant drop of IL-13 in PD-1-deficient T cells of the infected mice (Fig 3E and 3F). We also analyzed the mRNA of IL-4 and IL-13 in purified T cells from splenocytes of infected PD-1-deficient and WT animals, and we found a 1.6-fold and 2.4-fold decrease in IL-4 and IL-13 mRNA levels, respectively in the PD-1-deficient mice (Fig 3G). The IL-10 mRNA expression in T cells was significantly increased by PD-1 deficiency, but intracellular staining of IL-10 did not exhibit a significant increase in PD-1-deficient splenic CD4^+^ T cells (Fig 3E, 3F and 3G). Since IFN-γ production in T cells is crucial for viral clearance and parasite eradication [18,19], and IFN-γ is also associated with disease resistance to schistosomiasis [20,21], we decided to further analyze IFN-γ production in T cells of PD-1-deficient mice during chronic *S*. *japonicum* infection. Interestingly, we observed significantly increased IFN-γ in both the CD4^+^ and CD8^+^ T cells in the livers of PD-1-deficient mice 8 weeks after infection (Fig 3H and 3I). As IFN-γ is a major effector cytokine of Th1 cells, the differentiation of which is driven by the master transcription factor T-bet, we performed intracellular staining of T-bet protein in CD4^+^ T cells. As expected, in the livers of PD-1-deficient mice subjected to 8 weeks of *S*. *japonicum* infection, T-bet expression was significantly higher in CD4^+^ T cells than that in infected WT controls (Fig 3J and 3K). Our data showed that following *S*. *japonicum* infection, PD-1 deficiency significantly increased the number of T cells in the liver, and preferentially promoted IFN-γ production accompanied by decreased IL-4 production. Such data suggests that PD-1 plays an important role in balancing Th1 and Th2 differentiation in the setting of chronic *S*. *japonicum* infection.

### PD-1 deficiency does not significantly reduce parasite load or impact liver pathology in *S*. *japonicum*-infected mice

**Fig 4:**
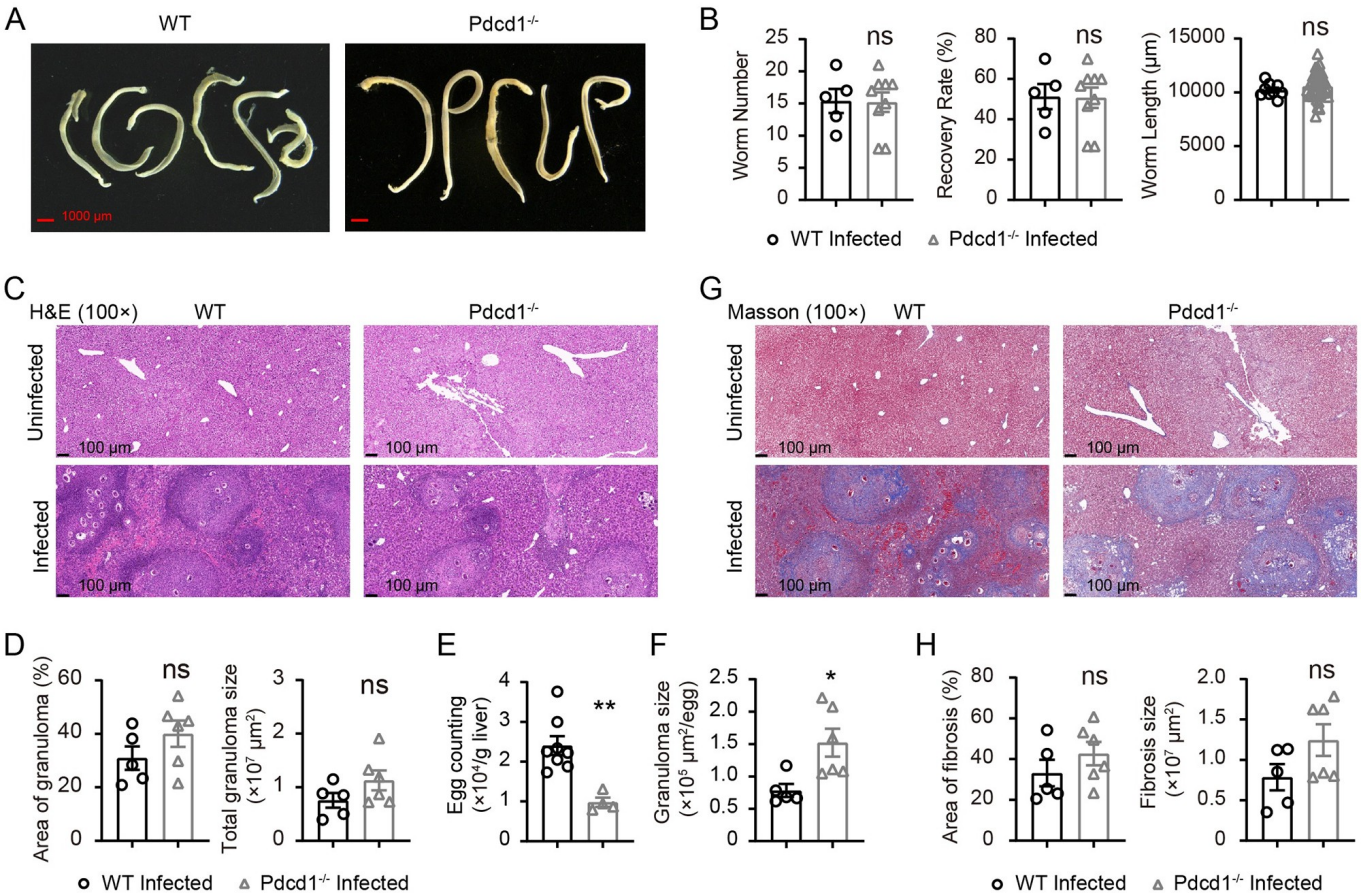
PD-1 deficiency does not significantly impact the pathogen load and pathology following *S*. *japonicum* infection. (A) Representative micrographs of adult *S*. *japonicum* parasites collected from portal and mesenteric veins of WT B6 mice using pump flush (left) and PD-1-deficient mice (right) 8 weeks after *S*. *japonicum* infection (scale bar, 1000 μm). (B) Number of worms, and recovery rate were compared between worms collected from WT B6 mice and PD-1-deficient mice 8 weeks after *S*. *japonicum* infection (WT mice, n = 5; KO mice, n = 9). Parasite length were compared between the same two groups of mice, namely WT infected and KO infected mice (worms from WT mice, n = 9; worms from KO mice, n = 36). (C-D) H&E staining of liver sections to compare granuloma between WT control mice (left) and PD-1-deficient mice (right) 8 weeks after *S*. *japonicum* infection (scale bar, 100 μm), and statistic comparisons of granuloma areas and sizes (WT mice, n = 5; KO mice, n = 6). (E) Counting of eggs in each gram of liver in WT control mice and PD-1-deficient mice 8 weeks after *S*. *japonicum* infection (WT mice, n = 8; KO mice, n = 4). (F) The granuloma size counted on the basis of single eggs in the H&E stained sections of the livers from WT control mice and PD-1-deficient mice 8 weeks after *S*. *japonicum* infection (WT mice, n = 5; KO mice, n = 6). (G-H) Masson staining of liver sections to compare liver fibrosis between WT B6 mice (left) and PD-1-deficient mice (right) 8 weeks after *S*. *japonicum* infection (scale bar, 100 μm), and statistic comparisons of fibrosis severity (WT mice, n = 5; KO mice, n = 6). For histological analyses, each data point was calculated from an independent animal. Data represent the mean ± s.e.m. Statistical significance was assessed by unpaired Student’s *t*-test or non-parametric unpaired Mann-Whitney test and indicated by ns, non-significant.

While we found reduced levels of IL-4 in CD4^+^ T cells from the infected livers of PD-1-deficient mice, we also observed a significant elevation in IFN-γ production by T cells. Therefore, we compared the parasite load and pathological grading of infected PD-1-deficient mice and WT control mice. We counted worms recovered from infected animals as described in our previous study [9]. Following 8 weeks of infection with 30 cercariae of *S*. *japonicum*, we counted the number of worms and compared worm length. The worm numbers, recovery rate and worm length were not significantly different between PD-1-deficient mice and WT controls following *S*. *japonicum* infection for 8 weeks (Fig 4A and 4B). Therefore, our experiments confirmed that loss of PD-1 did not result in eradication or lowering of pathogen load during *S*. *japonicum* infection.

It is also important that a balanced and controlled Th1 or Th2 response is critical for protective granuloma formation without excessive pathology during schistosomiasis [22]. In our experiments we first compared the Hematoxylin and Eosin (H&E) staining and immunohistochemistry (IHC) staining of liver sections to analyze granuloma and WBC infiltration using anti-mouse CD45. The total WBCs stained by anti-CD45 monoclonal antibody were not different between liver sections of PD-1-deficient mice and their WT controls following infection (S4A Fig), which was in line with the results of flow cytometric analyses to quantitate CD45^+^ WBCs in the liver. In the H&E staining experiments, we found that the granuloma areas were not significantly different between PD-1-deficient mice and WT controls following *S*. *japonicum* infection (Fig 4C and 4D). We also quantitatively analyzed the burden of parasite eggs in whole livers of PD-1-deficient mice and WT controls. Even though worm numbers and total granuloma areas were not significantly different, the egg counts in PD-1-deficient mice were significantly lower than those in WT controls mice (Fig 4E). When we quantified the granuloma size on the basis of single eggs, we found that granuloma size per egg in PD-1-deficient mice was significantly larger than that of the control mice (Fig 4F).

We further compared fibrosis severity between PD-1-deficient mice and WT controls following *S*. *japonicum* infection. Using Masson staining, we found comparable results between two groups of infected mice, which revealed that PD-1 deficiency did not alter fibrosis during chronic *S*. *japonicum* infection (Fig 4G and 4H). We also performed IHC staining of B cells and myeloid cells, and our data showed that PD-1 deficiency did not significantly impact the infiltration of B cells and myeloid cells in infected livers (S4B and S4C Fig). Our data show that in infected PD-1-deficient mice, pathogen load and parasite morphology were not significantly changed when compared to controls, while the parasite egg counts were significantly lower in PD-1-deficient mice. Moreover, the pathology grading of fibrosis and granuloma development was also not significantly altered by PD-1 deficiency during chronic *S*. *japonicum* infection, even though the granuloma size per egg was significantly higher in PD-1-deficient mice.

### PD-1 deficiency robustly boosts regulatory T cells in *S*. *japonicum* infected mice

In our study, PD-1 deficiency increased the absolute count of T cells in livers and spleens of *S*. *japonicum* infected mice, and such T cells in the liver expressed elevated IFN-γ in both CD4^+^ and CD8^+^ T subsets. However, the parasite loads as well as the severity of pathology were not reduced in PD-1-deficient mice. As Tregs play dominant roles in immune tolerance, we suspected that failure of the PD-1-deficient mice to eradicate or reduce parasites could be related to Treg-mediated immunosuppression. In our experiments using 30 cercariae of *S*. *japonicum* to infect WT mice for 8 weeks, we observed a significant drop of Foxp3 at both mRNA level (S5A Fig) and protein level in infected WT mice compared to uninfected WT controls (S5B and S5C Fig). Interestingly, such changes of Foxp3 protein cannot be detected in C57BL/6 wildtype mice in earlier phases of disease, such as 4 weeks after *S*. *japonicum* infection (S6A and S6B Fig). These data suggest that long-term infection can also result in dampened Tregs, which may involve PD-1 as an important regulator.

**Fig 5:**
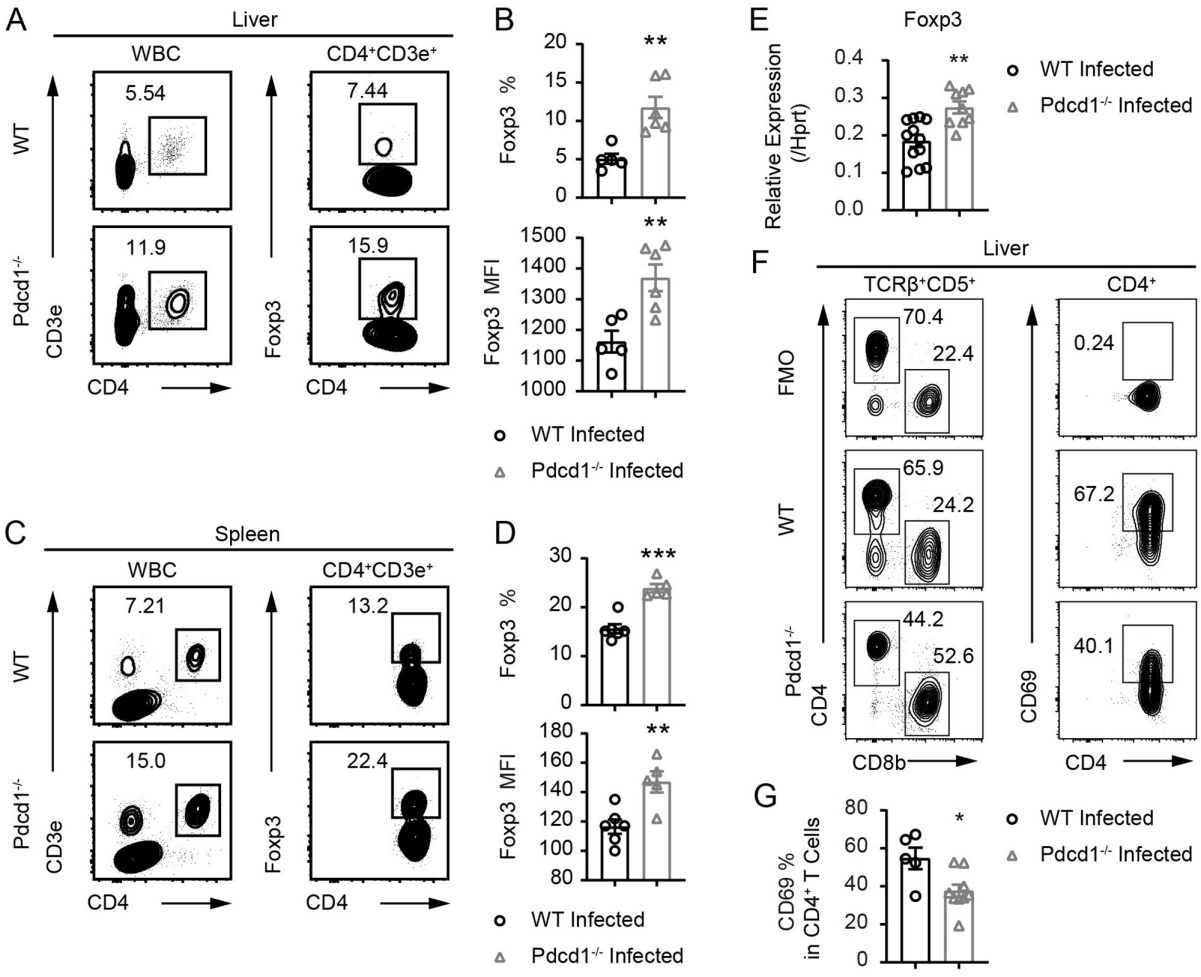
PD-1 deficiency robustly boosts regulatory T cells and inhibit T cell activation in *S*. *japonicum* infected mice. (A-B) Flow cytometric analyses of Foxp3 expression in CD4^+^ T cells in the livers of WT and PD-1-deficient *Pdcd1*^*−/−*^ mice infected with *S*. *japonicum* for 8 weeks (WT mice, n = 5; KO mice, n = 6). (C-D) Flow cytometric analyses of Foxp3 expression in CD4^+^ T cells in spleens of WT and PD-1-deficient mice infected with S. japonicum for 8 weeks (WT mice, n = 6; KO mice, n = 5). (E) qRT-PCR analyses of Foxp3 mRNAs in T cells purified from splenocytes of WT and PD-1-deficient mice infected with *S*. *japonicum* for 8 weeks (WT mice, n = 4; KO mice, n = 3; each sample analyzed in 3 replicates). (F-G) Flow cytometric analyses of CD69 expression in CD4^+^ T cells in the livers of WT and PD-1-deficient mice infected with *S*. *japonicum* for 8 weeks (WT mice, n = 5; KO mice, n = 9). Data represent the mean ± s.e.m. Statistical significance was assessed by unpaired Student’s *t*-test or non-parametric unpaired Mann-Whitney test and indicated by * *P*<0.05, ** *P*<0.01, *** *P*<0.001.

Next, we analyzed the immunosuppressive Tregs in PD-1-deficient mice and WT controls following *S*. *japonicum* infection. Strikingly, there was an obvious increase in Foxp3^+^ Tregs from livers of infected PD-1-deficient mice in comparison to infected WT controls 8 weeks after *S*. *japonicum* infection (Fig 5A and 5B). Such Treg increase and elevation of Foxp3 protein by PD-1 deficiency were also observed in the spleens of PD-1-deficient mice (Fig 5C and 5D). We compared the mRNA expression of Foxp3 in purified splenic T cells from infected mice and confirmed that the increase of Foxp3 observed at protein level was also occurring at transcriptional level in PD-1-deficient mice infected with *S*. *japonicum* (Fig 5E). In the schistosome-infected mice, PD-1-deficient T cells did not display a higher level of proliferation than their WT counterparts following T cell receptor (TCR) stimulation for 72 hours using coated anti-CD3 and soluble anti-CD28 for both CD4^+^ and CD8^+^ T cells (S7A and S7B Fig). We also analyzed activation marker CD69 in the CD4^+^ T cells from *S*. *japonicum* infected mice, and we found that in livers of PD-1-deficient mice, the frequency of CD69^+^CD4^+^ T cells had an approximate 1.5-fold drop as compared to the infected WT control mice (Fig 5F and 5G). Our data show that PD-1 deficiency does increase the frequency of Tregs and expression of Foxp3 protein during *S*. *japonicum* infection, which, therefore, can lead to inhibition of T cell activation *in vivo* and prevent host eradication of parasites.

### Depletion of natural regulatory T cells unleashes advantageous proliferation of PD-1-deficient T cells *in vitro* and *in vivo*

**Fig 6:**
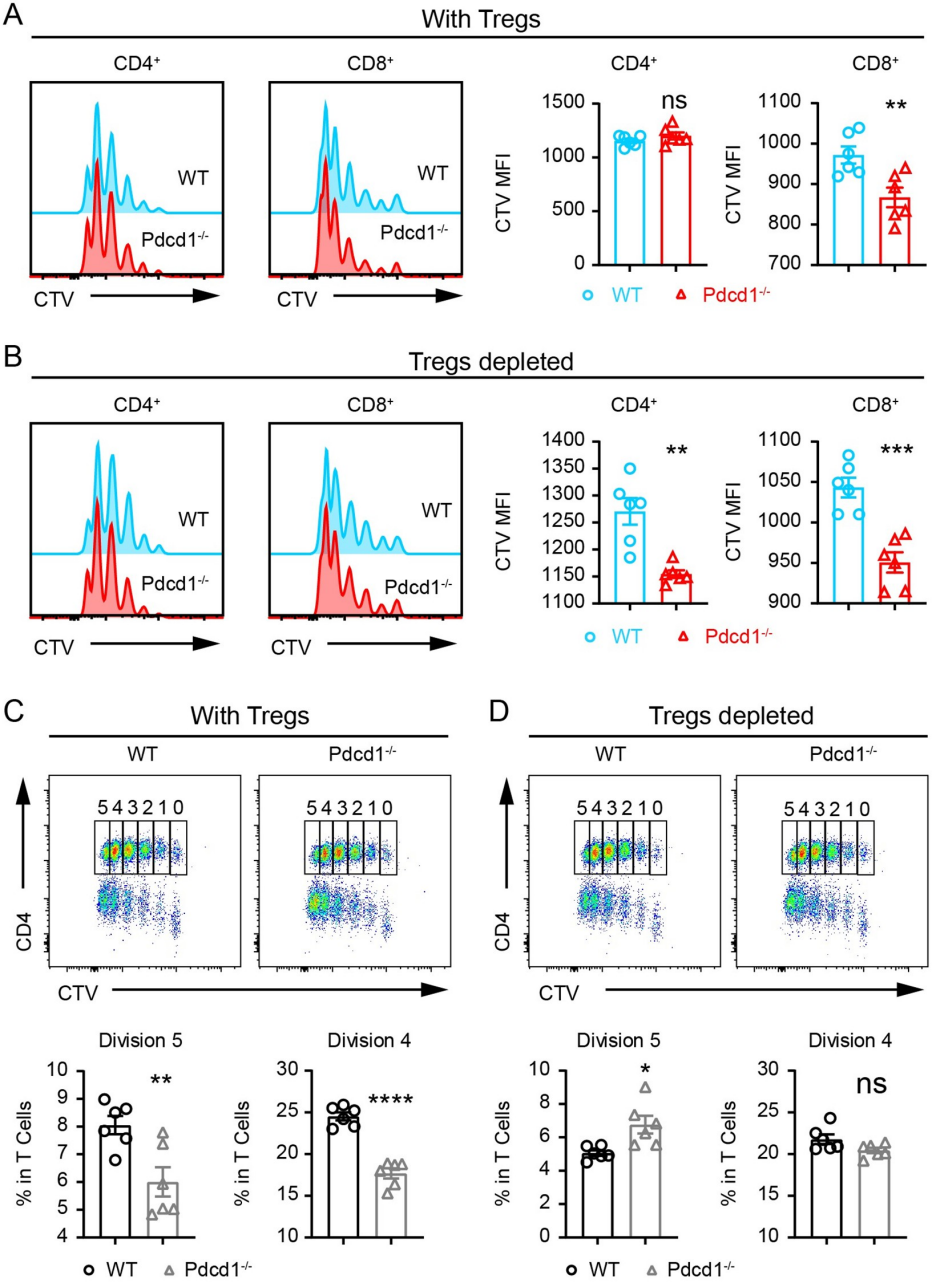
PD-1-deficient Tregs suppresses CD4^+^ T cell expansion upon TCR stimulation *in vitro*. (A) In T cells without Treg depletion, CTV dilution of CD4^+^ and CD8^+^ T cells purified from PD-1-deficient and WT mice was measured by FACS. Total T cells including Tregs were stimulated for 72 h with CD3 (3 μg/mL) and CD28 (1 μg/mL). (B) The MFI of CTV was compared between PD-1-deficient and WT CD4^+^ and CD8^+^ T cells with Treg depletion. (C) In T cells without Treg depletion, CTV dilution of CD4^+^ and CD8^+^ T cells purified from PD-1-deficient and WT mice was measured by FACS. T cells were stimulated for 72 h with CD3 (3 μg/mL) and CD28 (1 μg/mL). (D) The MFI of CTV was compared between PD-1-deficient and WT CD4^+^ and CD8^+^ T cells with Treg depletion. For each set of data 6 replicates were performed for statistic comparisons. Data represent the mean ± s.e.m. Statistical significance was assessed by unpaired Student’s *t*-test or non-parametric unpaired Mann-Whitney test and indicated by * *P*<0.05, ** *P*<0.01, *** *P*<0.001, **** *P*<0.0001, ns, non-significant.

In PD-1-deficient mice infected with *S*. *japonicum* for 8 weeks, Tregs were significantly higher in frequency and cell count, and PD-1-deficient T cells from infected mice did not display a higher level of proliferation than their WT counterparts following TCR stimulation. We further sought to compare T cell proliferation *in vitro* between uninfected PD-1-deficient mice and WT controls, in the absence of Tregs. We depleted Tregs in total T cells which were purified from lymph nodes, and Treg depletion was performed with biotinylated anti-CD25 antibody and magnetic streptavidin beads, which led to efficient depletion of Foxp3^+^ Tregs (S8A and S8B Fig). In the presence of Tregs, 72 h after TCR stimulation, CD4^+^ T cells exhibited comparable proliferation between PD-1-deficient T cells and WT controls and only PD-1-deficient CD8^+^ T cells had higher level of proliferation (Fig 6A). However, when the T cells received TCR stimulation under Treg depletion conditions, PD-1-deficient mice displayed a significantly higher level of proliferation for both CD4^+^ and CD8^+^ T cells compared to WT controls (Fig 6B). We further analyzed the CTV dilution and gated on the most divided cells, in which we found that depletion of Tregs significantly increased the frequency of PD-1-deficient CD4^+^ T cells. In the presence of Tregs, PD-1-deficient CD4^+^ T cells were lower in frequencies than WT controls among the most divided cells (Fig 6C), however Treg depletion significantly increased the frequencies of the PD-1-deficient CD4^+^ T cells (Fig 6D). Our *in vitro* data show that depletion of Tregs renders PD-1-deficient T cells outcompeting the WT T cells during TCR-induced proliferation.

**Fig 7:**
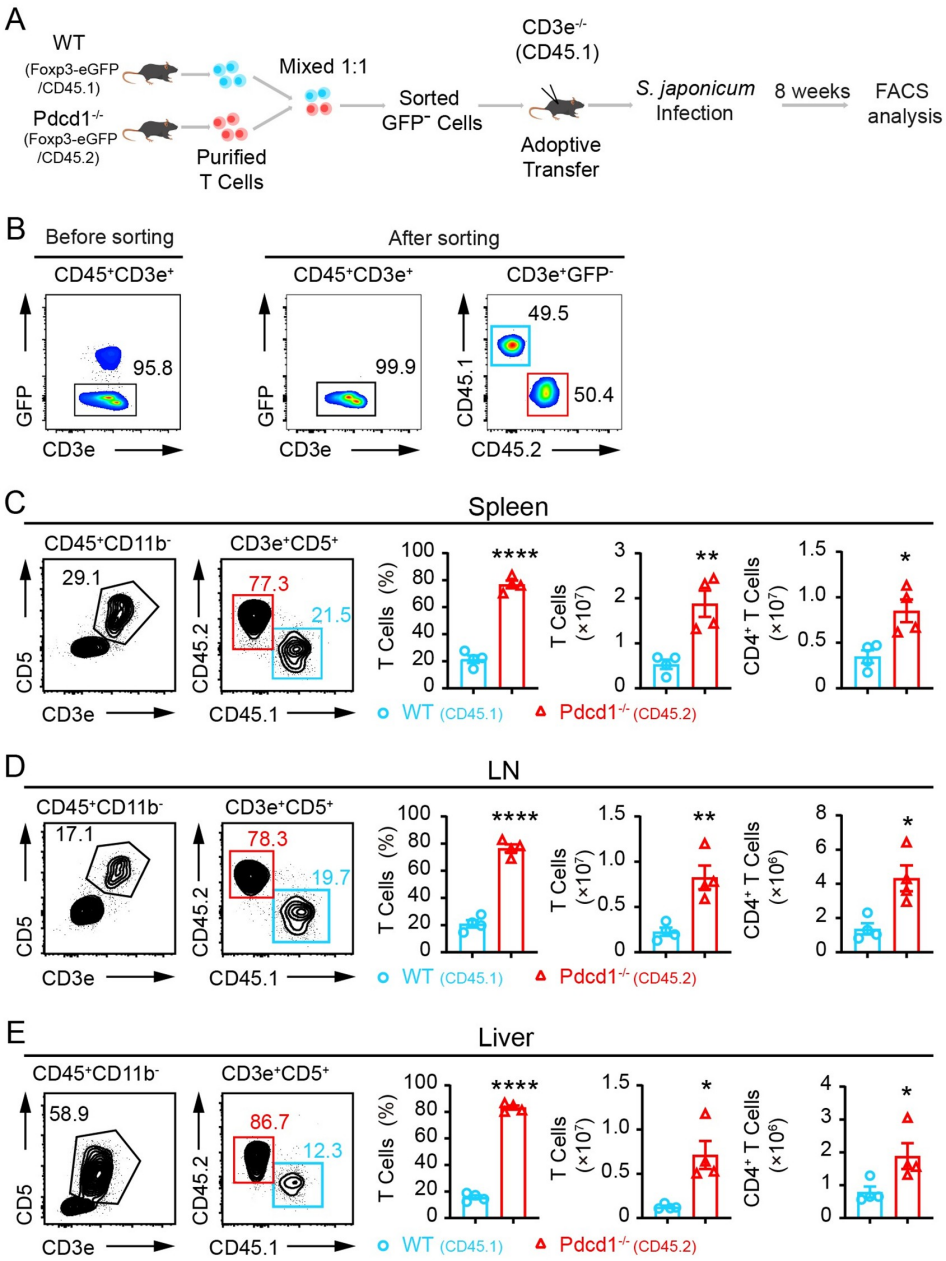
PD-1-deficient T cells dramatically outcompete wildtype T cells *in vivo* in the absence of natural Tregs during chronic *S*. *japonicum* infection. (A) Schematic presentation of experiments to compared WT and PD-1-deficient T cells *in vivo* in the absence of natural Tregs in *S*. *japonicum* infected mice. Purified total T cells from CD45.2^+^ Foxp3-eGFP^+^ *Pdcd1*^*−/−*^ mice, and CD45.1^+^ Foxp3-eGFP^+^ *Pdcd1*^*+/+*^ control mice were sorted to obtain GFP-negative T cells, which were co-transferred into CD45.1^+^ *CD3e*^*−/−*^ recipient mice with a 1:1 ratio and the recipient mice were infected with *S*. *japonicum* for 8 weeks. (B) FACS validation of cell sorting for GFP-negative T cells and mixture ratio between WT and PD-1-deficient cells before adoptive transfer into CD45.1^+^ *CD3e*^*−/−*^ recipient mice (n = 4). (C) Comparison of WT and PD-1-deficient T cells in spleens of recipient mice 8 weeks after *S*. *japonicum* infection. (D) Comparison of WT and PD-1-deficient T cells in lymph nodes of recipient mice 8 weeks after *S*. *japonicum* infection. (E) Comparison of WT and PD-1-deficient T cells in livers of recipient mice 8 weeks after *S*. *japonicum* infection. 4 samples of CD45.1^+^ *CD3e*^*−/−*^ recipient mice were analyzed for both WT and PD-1-deficient T cells. Data represent the mean ± s.e.m. Statistical significance was assessed by unpaired Student’s *t*-test or non-parametric unpaired Mann-Whitney test and indicated by * *P*<0.05, ** *P*<0.01, **** *P*<0.0001.

In further experiments, we compared WT and PD-1-deficient T cells *in vivo* in the absence of natural Tregs during *S*. *japonicum* infection. As systemic ablation of Tregs is fatal in immunocompetent adult mice, we used a T cell adoptive transfer model for chronic infection. We purified total T cells from CD45.2^+^ Foxp3-eGFP^+^ *Pdcd1*^*−/−*^ mice, and CD45.1^+^ Foxp3-eGFP^+^ *Pdcd1*^*+/+*^ control mice, and then we sorted GFP-negative T cells, and transferred the mixed T cells into *CD45*.*1*^*+*^ *CD3e*^*−/−*^ recipient mice which were then infected with *S*. *japonicum* (Fig 7A). Complete depletion of Tregs in GFP-negative T cells was validated by intracellular staining of Foxp3 before adoptive transfer (S9A and S9B Fig). We mixed the sorted GFP-negative T cells from PD-1-deficient and WT mice in a 1:1 ratio (Fig 7B). Following 8 weeks of *S*. *japonicum* infection, we analyzed T cells in spleens, lymph nodes and livers and compared cell counts of both total T cells and CD4^+^ T cells. In striking contrast to the 1:1 starting ratio, the PD-1-deficient T cells dramatically outcompeted WT controls after adoptive transfer and *S*. *japonicum* infection. There was a 3.6-fold increase of PD-1-deficient T cells in the spleens, a 3.7-fold increase in lymph nodes, and a 5.3-fold increase in livers in comparison to the co-transferred WT T cells (Fig 7C, 7D and 7E). In this T cell transfer model with complete natural Treg depletion, cell counts of PD-1-deficient CD4^+^ T cells in all analyzed organs including the spleen and lymph nodes were significantly higher than the WT controls. We also purified total T cells from WT and PD-1-deficient mice without depletion of Tregs, and performed adoptive transfer of equal numbers of purified T cells into CD45.1^+^ *CD3e*^*−/−*^ recipient mice, and then infected the recipient mice with *S*. *japonicum* for 8 weeks (S10A Fig). In this model, we did not deplete Tregs before adoptive transfer of PD-1-deficient and WT T cells, and we did not observe a significant change in the number of CD4^+^ T cells in the spleen and lymph nodes of the recipient mice (S10B and S10C Fig). In addition, we further compared PD-1-deficient CD45.2^+^ Tregs and WT CD45.2^+^ Tregs for their suppression of CD45.1^+^ conventional CD4^+^ T cells following TCR stimulation *in vitro*, and found that PD-1-deficient CD45.2^+^ Tregs exhibited significantly stronger suppression on expansion of CD4^+^ conventional T cells (S11 Fig). These data from *in vitro* and *in vivo* experiments show that when natural Tregs are depleted, the advantageous proliferation of PD-1-deficient T cells is unleashed, which suggests enhanced immunosuppression of PD-1-deficient Tregs.

### Enhanced immunosuppression of PD-1-deficient regulatory T cells is associated with increased adenosine receptors during *S*. *japonicum* infection

**Fig 8:**
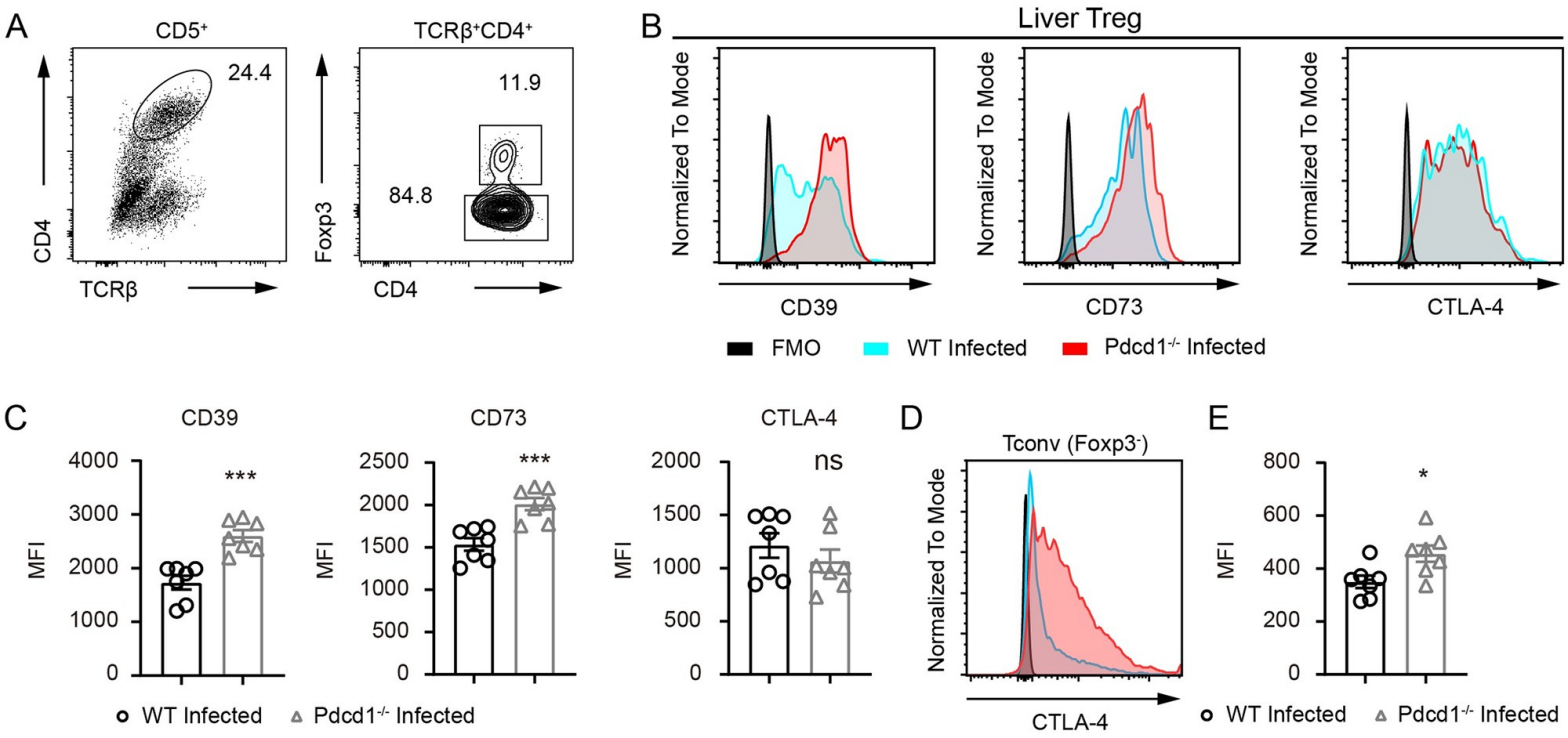
Excessive expression of immunosuppressive receptors by PD-1-deficient regulatory T cells. (A) FACS gating of Foxp3 positive and negative compartments in CD4^+^ T cells in the livers of WT and PD-1-deficient *Pdcd1*^*−/−*^ mice infected with *S*. *japonicum* for 8 weeks. (B-C) Flow cytometric analyses of CD39, CD73 and CTLA-4 in Foxp3^+^ Tregs in the livers of WT and PD-1-deficient mice infected with *S*. *japonicum* for 8 weeks (WT mice, n = 7; KO mice, n = 7). (D-E) Flow cytometric analyses of CTLA-4 in Foxp3 negative conventional CD4^+^ T cells in the livers of WT and PD-1-deficient mice infected with *S*. *japonicum* for 8 weeks (WT mice, n = 7; KO mice, n = 7). Data represent the mean ± s.e.m. Statistical significance was assessed by unpaired Student’s *t*-test or non-parametric unpaired Mann-Whitney test and indicated by * *P*<0.05, *** *P*<0.001, ns, non-significant.

Treg-mediated immunosuppression involves expression of cytokines and adenosine receptors. We analyzed surface expression of CD39 and CD73 in Tregs from the livers of PD-1-deficient and sufficient mice 8 weeks after *S*. *japonicum* infection by gating the Foxp3^+^ cells (Fig 8A). We analyzed the surface expression of CD39 and CD73 in Tregs and found that both immunosuppressive receptors were considerably increased by PD-1 deficiency in *S*. *japonicum* infected mice as measured by flow cytometry (Fig 8B and 8C). We found that loss of PD-1 did not change the expression of CTLA-4 expression in liver Tregs from infected mice (Fig 8B and 8C). However, when we analyzed the CTLA-4 expression in Foxp3 negative conventional T cells, we found a significant increase of in the expression of CTLA-4 in PD-1-deficient mice following 8 weeks of *S*. *japonicum* infection (Fig 8D). Taken together, our data from *in vitro* and *in vivo* experiments have shown that during chronic *S*. *japonicum* infection, deficiency results in obviously higher frequencies of Tregs and Treg-mediated immunosuppression, which is associated with higher expression levels of immunosuppressive receptors CD39 and CD73.

## Discussion

T cells are crucial for providing resistance against tumor and pathogens, as shown in numerous studies, including our previous study that confirmed T cells are crucial for the resistance of SD rats against schistosomes [8,9,23]. However, experimental evidence for modulating T cell responsiveness as a treatment or prevention of schistosomiasis is still very limited. PD-1 is a central regulator of T cell exhaustion, a state of T cell dysfunction that can occur during many chronic infections [8,24]. To date, experimental data from genetic animal models deficient in PD-1 and infection studies of schistosome parasites using such genetic models are still not available.

Schistosomiasis is a chronic disease associated with crippled adaptive immunity [6,25]. PD-1 induction in T cells of peripheral blood was found in patients infected with *S*. *japonicum* [17]. In this study, we have shown that as early as 4 weeks after *S*. *japonicum* cercariae challenge, liver CD4^+^ T cells express exaggerated amounts of PD-1. Specifically, we found that 50.6% of liver CD4^+^ T cells expressed PD-1 and 22.1% of splenic CD4^+^ T cells were PD-1 positive in infected mice. These data suggest that it is necessary to analyze T cell responses in the liver. Interestingly, even in the unisex infection experiments in which there were no parasitic eggs and granuloma formation, PD-1 was still notably induced in liver CD4^+^ T cells. To address the key question of whether PD-1 deficiency is sufficient to eradicate schistosomes in a susceptible host, such as C57BL/6 mouse, we established an experimental schistosomiasis model by infecting the PD-1-deficient mice and WT controls with *S*. *japonicum* cercariae. We observed a significant increase in the number of CD4^+^ and CD8^+^ T cells in the livers and spleens of infected PD-1-deficient mice. During schistosomiasis, schistosome eggs are massively produced by female parasites pairing with males and chronic infection causes a myeloid cell-dominated environment in the liver. In steady state, WT and PD-1-deficient mice had comparable frequencies of CD11b^+^ myeloid cells and T cell percentage was slightly higher (S12A and S12C Fig), however in the *S*. *japonicum* infected liver, less than 5% of the liver WBCs were T cells in infected WT mice while 2-fold more T cells in frequency were present in infected PD-1-deficient mice (S12B and S12D Fig), with the T cell to myeloid cell ratio extremely distorted after infection (S12E Fig). Such data show that adaptive immunity can be inhibited in the infected liver during schistosomiasis and PD-1 deficiency improves significantly the distorted T cell to myeloid cell ratio. It is well documented that schistosome eggs trigger a robust Type 2 immune response involving Th2 CD4^+^ T cells as one of the key players [26]. We also found that in splenic T cells of WT mice, infection obviously upregulated Th2 cytokines such as IL-4, IL-10, and IL-13 (S13 Fig). Unexpectedly, in both livers and spleens, we observed significantly decreased Th2 differentiation in T cells from PD-1-deficient mice following *S*. *japonicum* infection. Dampened Th2 differentiation in PD-1-deficient mice during schistosomiasis was in line with previous studies showing that Th2 differentiation can be suppressed by blockade of PD-1 [27]. Notably, blockade of PD-1 in cancer treatment results in beneficial therapeutic outcomes dependent on IFN-γ producing T cells [28], and dependence on T-bet to secrete IFN-γ is distinct for CD4^+^ and CD8^+^ T cells [29]. In infected PD-1-deficient mice, we observed a significant increase in IFN-γ production in CD4^+^ and CD8^+^ T cells, and higher T-bet expression in CD4^+^ T cells but not in CD8^+^ T cells, suggesting the existence of IFN-γ driving transcriptional program independent of T-bet during schistosomiasis.

Our data has shown that loss of PD-1 resulted in elevated numbers of liver T cells and increased IFN-γ secretion in T cells, which could be beneficial for the clearance of parasitic pathogens. Quite unexpectedly, our experiments showed that loss of PD-1 did not significantly impact the pathogen burden in mice infected with *S*. *japonicum* for 8 weeks. PD-1 deficiency also did not significantly change granuloma size or liver fibrosis after 8 weeks of *S*. *japonicum* infection, although the number of eggs was reduced significantly in PD-1-deficient mice and the granuloma size per egg concordantly increased. It is worth mentioning that in an infection model using *Toxoplasma gondii*, blockade of PD-L1, germline deletion of PD-1, or PD-1 deficiency in Tregs resulted in reduced pathogen-specific CD4^+^ T cell responses during infection [16]. Such discrepancies between different infection models suggest that the consequence of PD-1 deficiency is pathogen-dependent, revealing the complexity of modulating T cell responses via PD-1 to treat schistosomiasis.

Tregs are essential for immune tolerance and their roles in persistent infection and cancer are well appreciated. Notably blockade of PD-1 in different studies resulted in controversial findings concerning the impact on Tregs, in which some studies found PD-1 itself was required for Treg maintenance and PD-1 blockade led to suppression of Treg differentiation [30,31]. Other studies found the opposite results, showing that PD-1 blockade or loss of PD-1 could potently increase Treg differentiation [13,14], revealing the importance of analyzing PD-1 blockade or deficiency in multiple models involving different types of diseases and distinct genetic backgrounds. In WT mice, our previous experiments found that 6 weeks after *S*. *japonicum* infection, frequencies of Foxp3^+^ population were significantly higher in splenic CD4^+^ T cells of the susceptible C57BL/6 mice [9], and such phase-dependent Foxp3 expression may not exist in autoimmune disease model on the NOD background [13]. Interestingly in C57BL/6 mice, *S*. *japonicum* infection dynamically impacts Foxp3 expression in T cells, with no significant change 4 weeks after infection, but with a significant increase and decrease in Foxp3 expression at 6 and 8 weeks after infection, respectively.

As documented in previous studies, PD-1-deficient T cells expand more robustly following cognate antigen stimulation *in vivo*, and PD-1 blockade could increase T cell activation via TCR stimulation [32,33]. Indeed, when we stimulate *in vitro* the PD-1-deficient T cells, CD8^+^ T cells expanded significantly more in the presence or absence of Tregs. However, 72 h following TCR stimulation *in vitro* PD-1-deficient CD4^+^ T cell expansion only outcompeted WT controls when Tregs were depleted. Such findings were consistent with results from the competitive Treg suppression experiments in which PD-1-deficient CD45.2^+^ Tregs were more competitive than WT CD45.2^+^ Tregs in suppressing CD45.1^+^ conventional CD4^+^ T cells following TCR stimulation *in vitro*. It is important to note that in the absence of Tregs, T cells from PD-1-deficient mice intrinsically displayed a significantly higher level of proliferation for both CD4^+^ and CD8^+^ T cells when compared to WT controls. These data suggest that Treg-mediated immunosuppression prevents unfolding of the advantageous capacity for proliferation in PD-1-deficient T cells. It is also striking for us to find that in the complete absence of natural Tregs by sorting Foxp3-eGFP-negative T cells, there was a dramatic increase of PD-1-deficient T cells in all analyzed organs from the competitive co-transfer model infected by *S*. *japonicum* for 8 weeks. When PD-1-deficient T cells were adoptively transferred with intact Tregs, 8 weeks after *S*. *japonicum* infection, the increase in T cell numbers was not observed in the spleens and lymph nodes. Interestingly, in infected PD-1-deficient mice, CD69 expression in liver CD4^+^ T cells was significantly lower which also suggested immunosuppression by PD-1 deficiency. Taken together, the *in vitro* and *in vivo* data from our T cell transfer models show that Treg-mediated immunosuppression is excessively potent in PD-1-deficient T cells. To fully unleash the beneficial T cell response against schistosomes in PD-1-deficient mice, it still remains intriguing to remove the pathogen-responsive Tregs in immunocompetent animals.

During chronic schistosomiasis infected humans and model animals can develop severe liver fibrosis, leading to hypoxia in the liver [34,35]. Liver injury may cause upregulation of adenosine receptors, and ATP released from dead cells during chronic inflammation is also a potent inducer of CD39 and CD73-mediated immunosuppression [36,37], and adenosine receptors are known to prevent robust adaptive immune response [38–40]. In this study, we confirmed excessive amounts of CD39 and CD73 expression by PD-1-deficient Tregs in infected mice, as well as higher levels of CTLA-4 expression in conventional T cells from PD-1-deficient mice during infection, which could antagonize co-stimulatory signaling of T cells [41,42]. Increased expression of such canonical inhibitory molecules and excessive immunosuppression of PD-1-deficient Tregs may counteract any increased T cell presence or IFN-γ secretion in CD4^+^ and CD8^+^ T cells during *S*. *japonicum* infection. Taken together, our data indicate that the *S*. *japonicum* infection model established here provides unique scenarios under which function of PD-1 can be assessed, and also provides distinct mechanisms for T cell suppression during schistosomiasis.

## Materials and methods

### Ethics statement

All animal studies were reviewed and approved by the Ethics Review Committee of Xinxiang Medical University (Reference No. XYLL-2016S001).

### Animals

C57BL/6 mice were used for infection studies and controls, and mice used for genetic manipulation by genome editing were of C57BL/6N origin. The mice were purchased from Beijing Vital River Laboratory Animal Technology (China), a distributor of Charles River Laboratories. CD3e knockout mice and Foxp3-eGFP knock-in mice were kindly provided by the Bernard &Marie Malissen lab at Centre d’Immunologie de Marseille-Luminy in France. All mice were maintained in our Specific Pathogen-Free facility.

### Generation and genotyping of *Pdcd1*^*−/−*^ mice and *Foxp3-eGFP Pdcd1*^*−/−*^ mice

For CRISPR/Cas9-mediated gene deletion in C57BL/6 mice, sgRNAs targeting the mouse *Pdcd1* gene were designed by using the online CRISPOR tool (http://crispor.tefor.net/). *In vitro* transcription of Cas9 mRNA and sgRNAs, microinjections and transplant of embryos were performed as described in our previous studies [9,43]. The genomic DNA isolated from tails of F0 pups were used as a template for PCR screening to identify desirable mutant mice harboring bi-allelic deletion of the *Pdcd1* gene and then PCR products were subjected to Sanger sequencing. Primers for genotyping include: forward primer 5’-TCTGCTCCCCAATCTCTCACTAG-3’ and reverse primer 5’-GGTGTTCCTCCCCTCCAGTAATA-3’. The same genotyping protocol was applied to isolate homozygous *Foxp3-eGFP Pdcd1*^*−/−*^ mice.

### *Schistosoma japonicum* infection and parasite cercariae count

Each mouse was infected with 20 or 30 *S*. *japonicum* cercariae subcutaneously as described in our previous study [9]. In brief, the floating cercariae were transferred from flask to water droplet on cover glass, and such droplet as well as the cercariae were visualized using a Nikon SMZ745T microscope. Typically, 20 or 30 *S*. *japonicum* cercariae are transferred into 4 to 6 water droplets (~20 μL volume/droplet) on the cover glass. Infection of cercariae was performed with the aid of the cover glass, onto which droplets of water were loaded. Cercariae were transferred from 100 mL Erlenmeyer flask into the water droplets on the cover glass via a needle. Counting of cercariae was performed under the microscope for each droplet to know the exact number of cercariae in each droplet. After counting, the animal was stabilized and abdominal fur was removed and skin was cleaned with wet cotton. Finally, the cercariae-loaded cover glass was placed onto the skin of the animal for 20 minutes, allowing cercariae to penetrate.

### Parasite analysis, worm length quantification and parasite egg counting in the liver

Parasites were perfused through portal and mesenteric veins from infected mice 8 weeks after infection and the number of worms were recorded under Nikon SMZ745T microscope. Worm length were measured using NIS-Elements D 4.60.00 software. The recovery rate of worms was calculated as the ratio of adult worm number perfused/number of cercariae infected initially. Parasite egg counting in the liver was performed to determine the number of eggs per gram (EPG) of liver as described in a previous study [44]. Counting of the eggs was performed three times for each sample and the average was taken to calculate the liver EPG.

### Flow cytometry analysis

Immunophenotyping was performed in mice in steady state or after infection. Single cell suspension was prepared from spleens and livers of mice by mechanic dissociation using gentleMACS Dissociators (Miltenyi Biotec) with digestion enzymes (Sigma, DNase I, DN25; Sigma, Collagenase IV, C5138). Cells from spleens and livers were stained with monoclonal antibody mixes to analyze cell populations (S1 Table) and the labeled cells were acquired by flow cytometers (Thermo Fisher Scientific, Invitrogen Attune NxT Flow Cytometer and BD Biosciences, FACSCanto flow cytometry). The FACS data were analyzed by FlowJo software version 10.0.

### Intracellular staining of cytokines and Foxp3

The intracellular staining of cytokines was performed by stimulating T cells for 4 hours with 50 ng/ml PMA, 1 μg/ml ionomycin, and BD GolgiStop (BD Biosciences, Cat. 554724) before surface labeling with T cell markers. Subsequently, we determined cytokine production by treating the cells with BD Fix/Perm kit (BD Biosciences, Cat. 554722) and cells were treated with Perm/Wash Buffer (BD Biosciences, Cat. 554723) following the protocols provided by the reagent supplier. For Foxp3 staining, the cells collected *ex vivo* from livers, spleens and lymph nodes from mice in steady state or under infection were subjected to eBioscience Fix/Perm treatment and intranuclear staining (Invitrogen, Cat. 00-5523-00).

### *In vitro* stimulation of T cells

For PD-1 knockout mice phenotype validation was performed using lymphocytes from peripheral blood of WT and KO mice that were isolated with Lympholyte-Mammal Cell Separation Media (Cedarlane, CL5110) and then *in vitro* stimulation occurred using coated anti-CD3 (3 μg/mL, 145-2C11, BD Biosciences) and soluble anti-CD28 (1 μg/mL, 37.51, BD Biosciences).

### T cell purification, CTV labeling and adoptive transfer

T cells were purified by untouched enrichment via depletion of B cell and the majority of myeloid cells using biotinylated antibodies namely anti-CD19 (eBioscience, Biotin, 13-0193-86) and anti-CD11b (eBioscience, Biotin, MA5-17858) antibodies. The labeled cells were further incubated with Dynabeads M-280 Streptavidin kit (Invitrogen, 60210) following the protocol from manufacturer to negatively purify T cells. T cells purified for mRNA analyses were prepared in a similar manner but by positively enriching T cells using biotinylated CD5 antibody followed by cell isolation with magnetic beads from Dynabeads M-280 Streptavidin kit. Donor T cells were purified from infected mice (CD45.2^+^) and uninfected mice (CD45.1^+^CD45.2^+^ or noted as CD45.1/2 in schematic presentation) and were then used for adoptive cell transfer experiments with *CD3e*^***−/−***^ (CD45.1^+^) mice used as recipient mice. For the *in vivo* T cell proliferation assay, purified T cells from infected mice (CD45.2^+^) and uninfected mice (CD45.1^+^CD45.2^+^) were mixed 1:1 and labeled with CTV (CellTrace Violet Cell Proliferation Kit, Invitrogen, C34557) for 20 minutes at 37°C followed by quenching with FACS buffer. Cells were washed twice in PBS, then 8 million cells were transfused per mouse via retro-orbital injection. Mice were sacrificed 5 days after transfusion, and T cells from lymph nodes were analyzed by flow cytometry.

### Treg depletion for adoptive transfer and *in vitro* TCR stimulation

To deplete Tregs in CD45.2^+^ Foxp3-eGFP^+^ *Pdcd1*^*−/−*^ mice and CD45.1^+^ Foxp3-eGFP^+^ *Pdcd1*^*+/+*^ control mice for T cell transfer, T cells were first purified by untouched mouse T cell Dynabeads (Invitrogen, 11413) and then Foxp3-eGFP^+^ T cells on WT or PD-1-deficient background were sorted by gating GFP-positive and negative cells. Cell sorting experiments were performed by using BD FACSAria Fusion cell sorter. Two million GFP-negative T cells from CD45.2^+^ Foxp3-eGFP^+^ *Pdcd1*^*−/−*^ mice and CD45.1^+^ Foxp3-eGFP^+^ *Pdcd1*^*+/+*^ were used for a 1:1 ratio mixture for each recipient mouse. For *in vitro* TCR stimulation of Treg-depleted WT and PD-1-deficient T cells, purified total T cells were labeled with biotinylated anti-CD25 antibody (Cat.13-0252-86, Clone eBio7D4, eBioscience), which was followed by 3 washes with cold FACS buffer, and addition of Dynabeads M-280 Streptavidin beads (Invitrogen, 60210) to deplete Tregs.

### Real-time PCR

Total RNA was extracted from splenic T cells by using MiniBEST Universal RNA Extraction Kit (TaKaRa, 9767) for mRNA preparation. cDNA samples were synthesized with RevertAid First Strand cDNA Synthesis Kit (Thermo Fisher Scientific, K1622) and used as templates for RT-qPCR. RT-qPCR reactions were performed with TB Green Premix Ex Taq (TaKaRa, RR420A) and run on a 7500/7500 Fast Real-Time PCR System (Thermo Fisher Scientific, Applied Biosystems). Relative gene expression was calculated by the 2(−ΔCT) method using *Hprt* gene as housekeeping gene controls. Gene-specific primers are listed in S2 Table.

### Liver pathology analyses of infected mice

Liver samples were collected and immediately fixed in 4% paraformaldehyde. Liver sections were stained with H&E and Masson’s trichrome for analysis of liver granulomas and liver fibrosis, respectively. CD45 (Servicebio, GB11066), CD11b (Servicebio, GB11058) and CD19 (Servicebio, GB11061-1) staining were performed for immunohistochemical analysis, according to standard procedures as our previously study described[9]. Images were scanned by Pannoramic MIDI II (3D HISTECH) and analyzed with HALO image analysis software (Indica Labs) and CaseViewer software (3D HISTECH).

### Statistical analysis

All data were presented as means ± s.e.m and GraphPad Prism software (version 8.0) was used for data analysis. Statistical significance was assessed by parametric (unpaired Student’s *t-*test) and nonparametric (Mann–Whitney test) methods when two groups were compared. *, *P<*0.05; **, *P<*0.01; ***, *P<*0.001; ****, *P<*0.0001, ns, non-significant.

## Supporting information

S1 Table Antibodies and fluorescent dyes used for flow cytometric analysis in this study. (XLSX)

S2 Table Primers used for RT-PCR in this study. (XLSX)

S1 Data Excel file containing the numerical data and statistical analyses for Figure panels 1A, 1B, 1C, 1D, 1I, 2D, 2F, 2H, 3B, 3D, 3F, 3G, 3I, 3K, 4B, 4D, 4E, 4F, 4H, 5B, 5D, 5E, 5G, 6A, 6B, 6C, 6D, 7C, 7D, 7E, 8C, 8E, S1A, S1B, S2B, S2C, S3B, S3D, S4A, S4B, S4C, S5A, S5C, S6B, S7B, S10B, S10C, S11C, S11D, S12C, S12D, S12E and S13A in separate sheets.
(XLSX)

S1 Fig PD-1 induction in liver and spleen T cells during early stage of *S*. *japonicum* infection. 4 weeks after *S*. *japonicum* infection with 30 cercariae, (A) liver T cells including CD4^+^ and CD8^+^ T cells were analyzed for PD-1 expression, and (B) parallel analyses were performed for CD4^+^ and CD8^+^ T cells in the spleen. (Infected mice, n = 6; Uninfected mice, n = 6). Data represent the mean ± s.e.m. Statistical significance was assessed by unpaired Student’s *t*-test or non-parametric unpaired Mann-Whitney test and indicated by* *P*<0.05, *** *P*<0.001, **** *P*<0.0001.
(TIF)

S2 Fig Unisex schistosome infection and PD-1 expression in liver T cells. 8 weeks after unisex *S*. *japonicum* infection with 30 cercariae PD-1 expression was analyzed in the liver T cells, in the absence of parasitic eggs. (A) H&E staining and Masson staining of liver sections from uninfected and infected mice, representative results from two group of animals namely the uninfected controls and unisex worm infected mice. (B-C) PD-1 expression in the liver of T cells by flow cytometry using FMO control for gating. The frequencies of PD-1 expressing cells in the CD4^+^ and CD8^+^ T cells of the liver were compared (B), and the histogram and MFI were also compared between uninfected and unisex worm infected animals. (Infected mice, n = 10; Uninfected mice, n = 5). Data represent the mean ± s.e.m. Statistical significance was assessed by unpaired Student’s *t*-test or non-parametric unpaired Mann-Whitney test and indicated by *** *P*<0.001, **** *P*<0.0001, ns, non-significant.
(TIF)

S3 Fig T cells in spleens and livers of WT and PD-1-deficient mice in steady state. (A) and (B) In the spleen counts of total T cells, CD4/CD8^+^ subsets and frequencies of effector memory gated by CD44^high^ CD62L^low^ cells between WT (n = 7) and PD-1 (n = 7) deficient mice in steady state. (C) and (D) In the liver counts of total T cells, CD4/CD8^+^ subsets and frequencies of effector memory gated by CD44^high^ CD62L^low^ cells between WT (n = 7) and PD-1 (n = 7) deficient mice in steady state. Data represent the mean ± s.e.m. Statistical significance was assessed by unpaired Student’s *t*-test or non-parametric unpaired Mann-Whitney test and indicated by * *P*<0.05, **** *P*<0.0001, ns, non-significant.
(TIF)

S4 Fig PD-1 deficiency does obviously not change B cell and CD11b^+^ myeloid cell IHC staining in livers following *S*. *japonicum* infection. (A) CD45 staining of liver sections from infected WT and PD-1-deficient *Pdcd1*^*−/−*^ mice, and CD45^+^ area shown as percentage measured from CD45 stained liver sections using HALO image analysis software (WT mice, n = 5; KO mice, n = 6). (B) CD19 staining of liver sections from infected WT and PD-1-deficient mice, and CD19^+^ area shown as percentage measured from CD19 stained liver sections using HALO image analysis software (WT mice, n = 5; KO mice, n = 6). (C) CD11b staining of liver sections from infected WT and PD-1-deficient mice, and CD11b^+^ area shown as percentage measured from CD11b stained liver sections using HALO image analysis software (WT mice, n = 5; KO mice, n = 6). Original magnification, ×100; scale bar, 100 μm. Data represent the mean ± s.e.m. Statistical significance was assessed by unpaired Student’s *t*-test or non-parametric unpaired Mann-Whitney test and indicated by ns, non-significant.
(TIF)

S5 Fig Foxp3 expression in wildtype mice with 8 weeks of *S*. *japonicum* infection. (A) mRNA expression of Foxp3 in T cells of the spleens from WT mice with or without *S*. *japonicum* infection. mRNA expression was analyzed with 12 replicates for each group of mice. (B) and (C) FACS analysis of splenic Tregs by Foxp3 staining using WT mice with or without *S*. *japonicum* infection (uninfected, n = 7; infected, n = 5). For the infected group, WT mice were infected with *S*. *japonicum* for 8 weeks. Data represent the mean ± s.e.m. Statistical significance was assessed by unpaired Student’s *t*-test or non-parametric unpaired Mann-Whitney test and indicated by * *P*<0.05, *** *P*<0.001.
(TIF)

S6 Fig Regulatory T cells are not obviously induced during early phase of *S*. *japonicum* infection in C57BL/6 WT mice. (A-B) Flow cytometric analyses of Foxp3 expression in CD4^+^ T cells in spleens of C57BL/6 uninfected controls and mice infected with 20 S. japonicum cercariae for 4 weeks (WT mice, n = 6; KO mice, n = 6). Data represent the mean ± s.e.m. Statistical significance was assessed by unpaired Student’s *t*-test or non-parametric unpaired Mann-Whitney test and indicated by ns, non-significant.
(TIF)

S7 Fig Proliferation of T cells from WT and PD-1-deficient *Pdcd1*^*−/−*^ mice following *S*. *japonicum* infection. (A-B) Flow cytometric analyses of CTV dilution in CD4^+^ and CD8^+^ T cells collected from spleens of WT and PD-1-deficient *Pdcd1*^*−/−*^ mice, both of which were infected with *S*. *japonicum* for 8 weeks. T cells were stimulated *in vitro* by treatment with coated anti-CD3 (3 μg/mL) and soluble anti-CD28 (1 μg/mL) antibodies for 72 h. Statistic comparisons were made between two group of mice for MFI of CTV in both CD4^+^ and CD8^+^ T cells (WT mice, n = 6; KO mice, n = 6). Data represent the mean ± s.e.m. Statistical significance was assessed by unpaired Student’s *t*-test or non-parametric unpaired Mann-Whitney test and indicated by ns, non-significant.
(TIF)

S8 Fig T cell purification and Treg depletion for *in vitro* TCR stimulation using WT and PD-1-deficient *Pdcd1*^*−/−*^ mice. (A) Total cells from lymph nodes from two group of mice were subjected to purification with Dynabeads respectively, purity of T cells from each genotype were verified by FACS. (B) Purified WT and PD-1-deficient total T cells were incubated with anti-CD25 biotinylated antibody followed by washes and incubation with magnetic streptavidin beads for depletion of CD25^+^ cells. Representative FACS data of Foxp3 intracellular in purified T cells before and after Treg depletion in WT and PD-1-deficient T cells.
(TIF)

S9 Fig Complete Treg depletion by sorting Foxp3-eGFP-negative T cells for adoptive transfer. (A-B) Foxp3 staining was tested for sorted Foxp3-eGFP-negative and positive T cells before adoptive transfer. 2 × 10^6^ sorted GFP-negative T cells from CD45.2^+^ Foxp3-eGFP^+^ *Pdcd1*^*−/−*^ mice and CD45.1^+^ Foxp3-eGFP^+^ *Pdcd1*^*+/+*^ respectively were mixed in a 1:1 ratio for each recipient mouse.
(TIF)

S10 Fig T cell count in CD45.1^+^ *CD3e*^−/−^ recipient mice after adoptive transfer of T cells (with Tregs) from WT and PD-1-deficient mice and *S*. *japonicum* infection. (A) Schematic presentation of the experiments involving total T cell purification, and transfer of equal number of total T cells (with Tregs) into CD45.1^+^ *CD3e*^−/−^ mice before *S*. *japonicum* infection. (B) Flow cytometric analyses of T cells from WT and PD-1-deficient T cells collected from the spleen of recipient mice 8 weeks after *S*. *japonicum* infection. Total T cell counts and CD4^+^ T cell counts were presented between two group of adoptively transferred mice. (C) Flow cytometric analyses of T cells from WT and PD-1-deficient T cells collected from the livers of recipient mice after *S*. *japonicum* infection. Total T cell counts and CD4^+^ T cell counts were presented between two group of adoptively transferred mice. The two groups of recipient mice (WT group, n = 5; KO group, n = 5) adoptively transferred with purified WT and PD-1-deficient T cells, respectively were infected with *S*. *japonicum* for 8 weeks before FACS analyses. 8×10^6^ purified WT and PD-1-deficient T cells (with Tregs) were respectively transferred into each CD45.1^+^ *CD3e*^−/−^ recipient mice before *S*. *japonicum* infection for 8 weeks. Data represent the mean ± s.e.m. Statistical significance was assessed by unpaired Student’s *t*-test or non-parametric unpaired Mann-Whitney test and and indicated by * *P*<0.05, ns, non-significant.
(TIF)

S11 Fig PD-1-deficient CD45.2^+^ Tregs are more competitive than WT CD45.2^+^ Tregs in suppressing CD45.1^+^ conventional CD4 T cells following TCR stimulation *in vitro*. (A) Schematic workflow of the experiment which includes multiple steps such as isolation of conventional CD4^+^ T cell from CD45.1 congenic mice by untouched mouse CD4 T cell Dynabeads supplemented with rat anti-mouse CD25 monoclonal antibody, cell sorting of two types of Tregs from PD-1 knockout and WT mice, mixture of WT or PD-1-deficient Tregs and CD4 Tconv cells in different ratios, TCR stimulation (anti-CD3 antibody 1 μg/mL and anti-CD28 antibody 1 μg/mL) *in vitro* and cell division analysis. (B) Representative FACS analyses of T cell divisions by gating CTV labelled CD45.1^+^ CD4 Tconv with TCR stimulation for 72 h, in the presence of WT or PD-1-deficient Tregs at the Treg:Tconv ratio of 1:4. (C) Statistic comparisons of CTV fluorescence attenuation between CD45.1^+^ CD4 Tconv cells, which were co-cultured with WT or PD-1-deficient Tregs at the Treg:Tconv ratios of 0:1, 1:2, 1:4 and 1:8. Empty squares represent data points of CTV levels from CD45.1^+^ CD4 Tconv cells without co-culture of Tregs. Solid blue dots represent data points of CTV levels from CD45.1^+^ CD4 Tconv cells co-cultured with WT Tregs, and red triangles represent data points of CTV levels from CD45.1^+^ CD4 Tconv cells co-cultured with PD-1 knockout Tregs. (D) Percentage of cell divisions analyzed by gating CD45.1^+^ CD4 Tconv cells with TCR stimulation for 72 h. Division 4 and 5 indicate cells undergoing more divisions and faster cell expansion, and Division 1 and 2 indicate cells with less divisions and slower cell expansion. Among two group of CD45.1^+^ CD4 Tconv cells co-cultured with either WT Tregs or PD-1 knockout Tregs, lower percentage of fast-growing cells indicates stronger Treg-mediated suppression. n = 5 repeats for all the conditions. Data represent the mean ± s.e.m. Statistical significance was assessed by unpaired Student’s *t*-test or non-parametric unpaired Mann-Whitney test and indicated by * *P*<0.05, ** *P*<0.01, ns, non-significant.
(TIF)

S12 Fig Frequencies of T cells and myeloid cells in the liver of WT and PD-1-deficient mice with or without *S*. *japonicum* infection. (A) In livers of un-infected mice, FACS gating for frequencies of CD11b^+^ myeloid cells and CD3^+^ T cells involving both WT and PD-1-deficient animals. (B) In livers of *S*. *japonicum* infected mice, FACS gating for frequencies of CD11b^+^ myeloid cells and CD3^+^ T cells involving both WT and PD-1-deficient animals (WT, n = 7; KO, n = 7). (C) In livers of un-infected mice, statistic comparisons for frequencies of CD11b^+^ myeloid cells and CD3^+^ T cells involving both WT and PD-1-deficient animals. (D) In livers of *S*. *japonicum* infected mice, statistic comparisons for frequencies of CD11b^+^ myeloid cells and CD3^+^ T cells involving both WT and PD-1-deficient animals (WT, n = 5; KO, n = 9). (E) Ratio of CD3^+^ cells to CD11b^+^ cells in the infected and un-infected animals, the same data points from (C) and (D) were used for calculation of the ratio. Data represent the mean ± s.e.m. Statistical significance was assessed by unpaired Student’s *t*-test or non-parametric unpaired Mann-Whitney test and indicated by * *P*<0.05, ** *P*<0.01, ns, non-significant.
(TIF)

S13 Fig mRNA expression of Th2 cytokines in splenic T cells of wildtype mice with or without *S*. *japonicum* infection. For the infected group, WT mice were infected with *S*. *japonicum* for 8 weeks. Each sample was analyzed with 9 replicates. Data represent the mean ± s.e.m. Statistical significance was assessed by unpaired Student’s *t*-test or non-parametric unpaired Mann-Whitney test and indicated by ** *P*<0.01, *** *P*<0.001, **** *P*<0.0001.
(TIF)
