# Supplementary figures and images for: Excessive immunosuppression by regulatory T cells antagonizes T cell response to schistosome infection in PD-1-deficient mice
Source: PLoS Pathog. 2022 Jun 6;18(6):e1010596. doi: 10.1371/journal.ppat.1010596 (PMC9203022; doi:10.1371/journal.ppat.1010596)

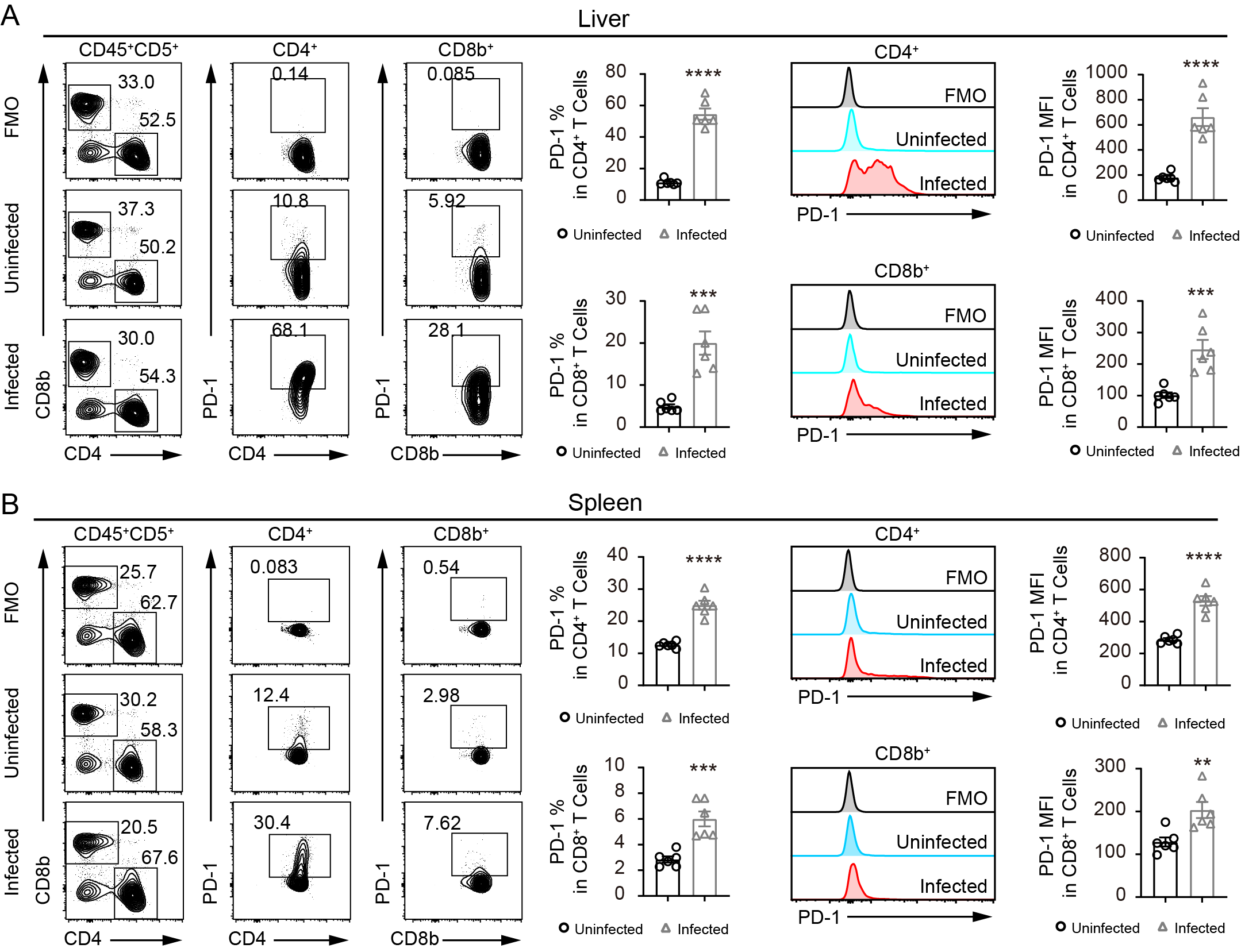

Supplement: S1 Fig — 4 weeks after S. japonicum infection with 30 cercariae, (A) liver T cells including CD4+ and CD8+ T cells were analyzed for PD-1 expression, and (B) parallel analyses were performed for CD4+ and CD8+ T cells in the spleen. (Infected mice, n = 6; Uninfected mice, n = 6). Data represent the mean ± s.e.m. Statistical significance was assessed by unpaired Student’s t-test or non-parametric unpaired Mann-Whitney test and indicated by* P<0.05, *** P<0.001, **** P<0.0001. (TIF) [file ppat.1010596.s004.tif]

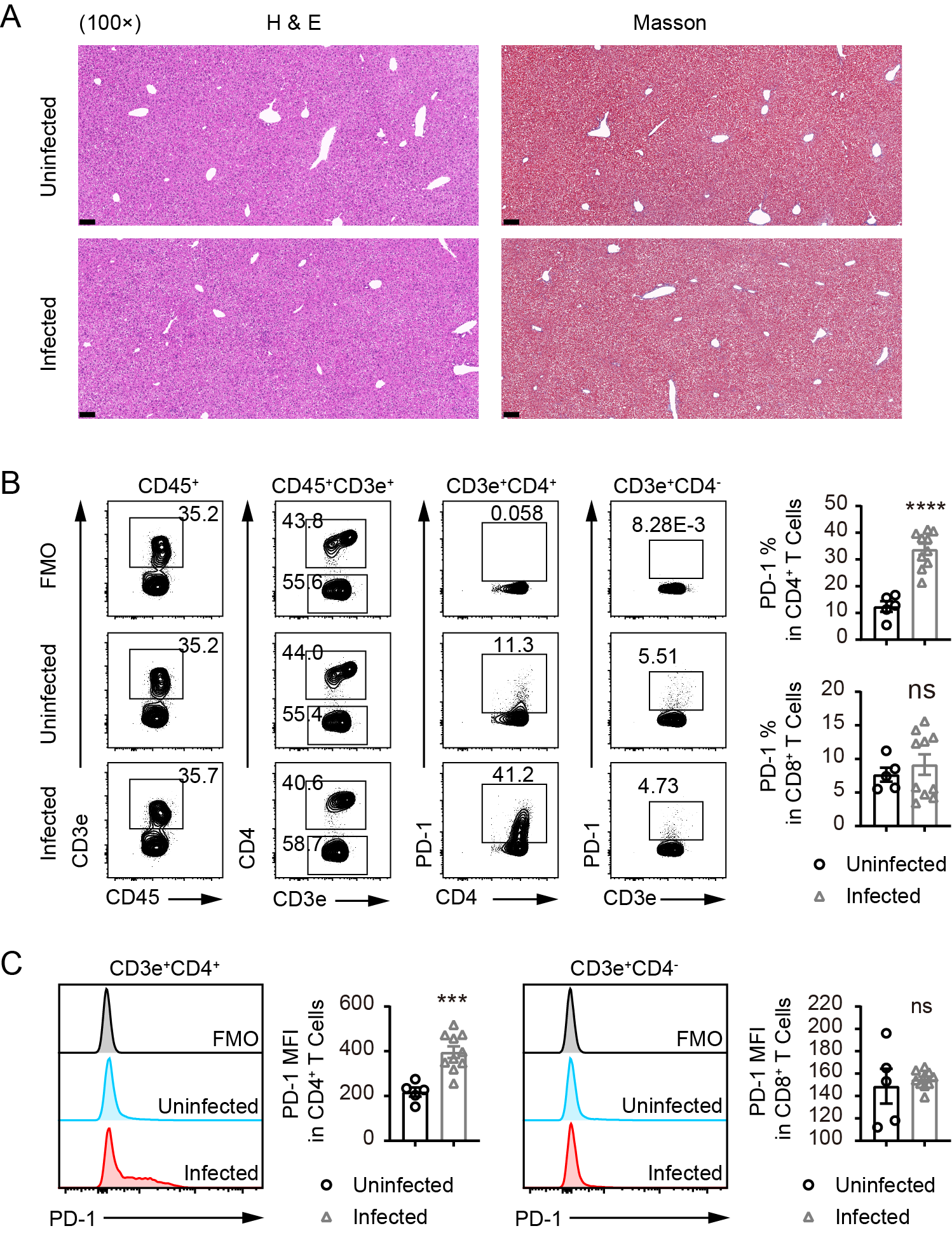

Supplement: S2 Fig — 8 weeks after unisex S. japonicum infection with 30 cercariae PD-1 expression was analyzed in the liver T cells, in the absence of parasitic eggs. (A) H&E staining and Masson staining of liver sections from uninfected and infected mice, representative results from two group of animals namely the uninfected controls and unisex worm infected mice. (B-C) PD-1 expression in the liver of T cells by flow cytometry using FMO control for gating. The frequencies of PD-1 expressing cells in the CD4+ and CD8+ T cells of the liver were compared (B), and the histogram and MFI were also compared between uninfected and unisex worm infected animals. (Infected mice, n = 10; Uninfected mice, n = 5). Data represent the mean ± s.e.m. Statistical significance was assessed by unpaired Student’s t-test or non-parametric unpaired Mann-Whitney test and indicated by *** P<0.001, **** P<0.0001, ns, non-significant. (TIF) [file ppat.1010596.s005.tif]

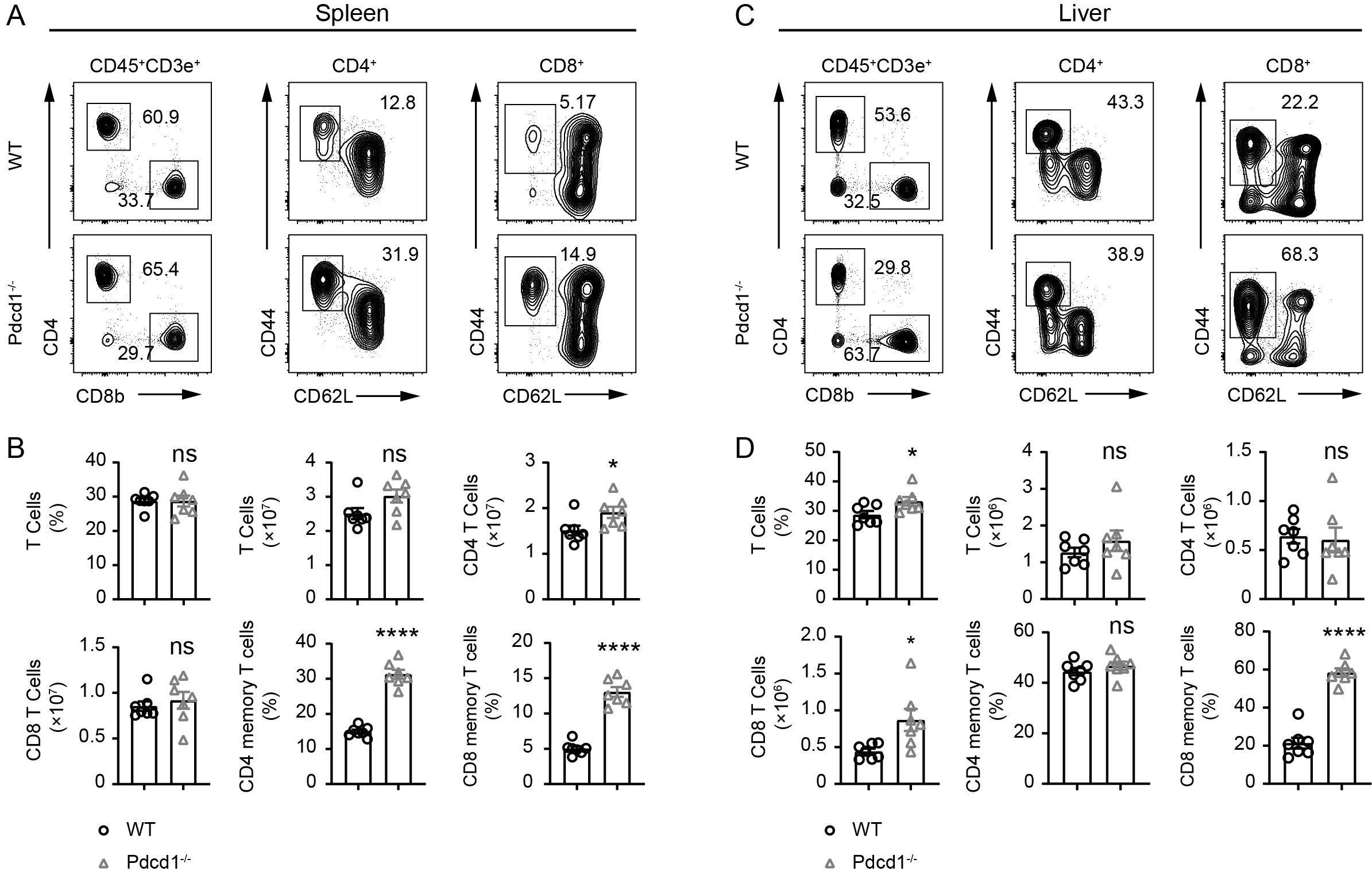

Supplement: S3 Fig — (A) and (B) In the spleen counts of total T cells, CD4/CD8+ subsets and frequencies of effector memory gated by CD44high CD62Llow cells between WT (n = 7) and PD-1 (n = 7) deficient mice in steady state. (C) and (D) In the liver counts of total T cells, CD4/CD8+ subsets and frequencies of effector memory gated by CD44high CD62Llow cells between WT (n = 7) and PD-1 (n = 7) deficient mice in steady state. Data represent the mean ± s.e.m. Statistical significance was assessed by unpaired Student’s t-test or non-parametric unpaired Mann-Whitney test and indicated by * P<0.05, **** P<0.0001, ns, non-significant. (TIF) [file ppat.1010596.s006.tif]

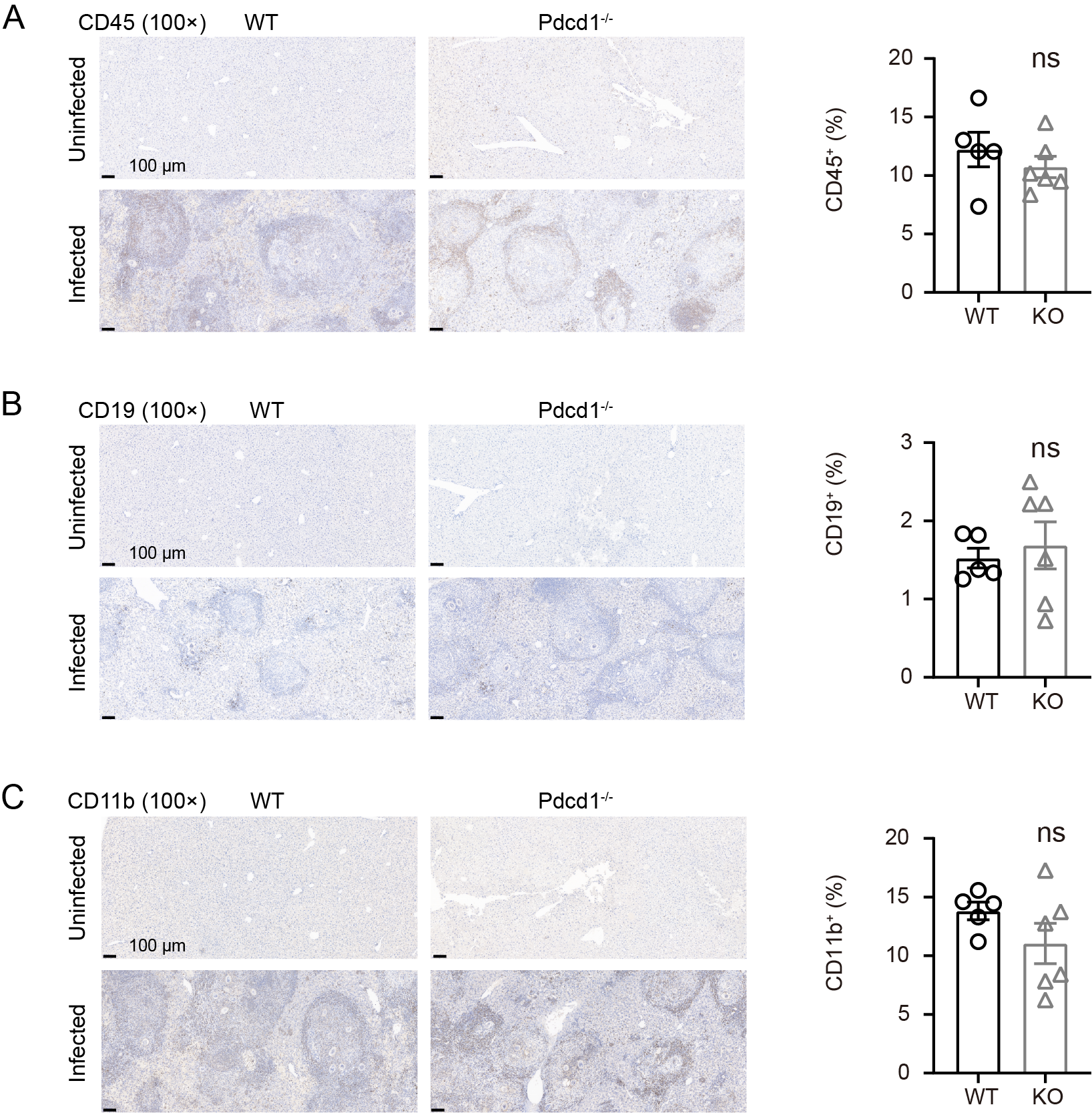

Supplement: S4 Fig — (A) CD45 staining of liver sections from infected WT and PD-1-deficient Pdcd1−/− mice, and CD45+ area shown as percentage measured from CD45 stained liver sections using HALO image analysis software (WT mice, n = 5; KO mice, n = 6). (B) CD19 staining of liver sections from infected WT and PD-1-deficient mice, and CD19+ area shown as percentage measured from CD19 stained liver sections using HALO image analysis software (WT mice, n = 5; KO mice, n = 6). (C) CD11b staining of liver sections from infected WT and PD-1-deficient mice, and CD11b+ area shown as percentage measured from CD11b stained liver sections using HALO image analysis software (WT mice, n = 5; KO mice, n = 6). Original magnification, ×100; scale bar, 100 μm. Data represent the mean ± s.e.m. Statistical significance was assessed by unpaired Student’s t-test or non-parametric unpaired Mann-Whitney test and indicated by ns, non-significant. (TIF) [file ppat.1010596.s007.tif]

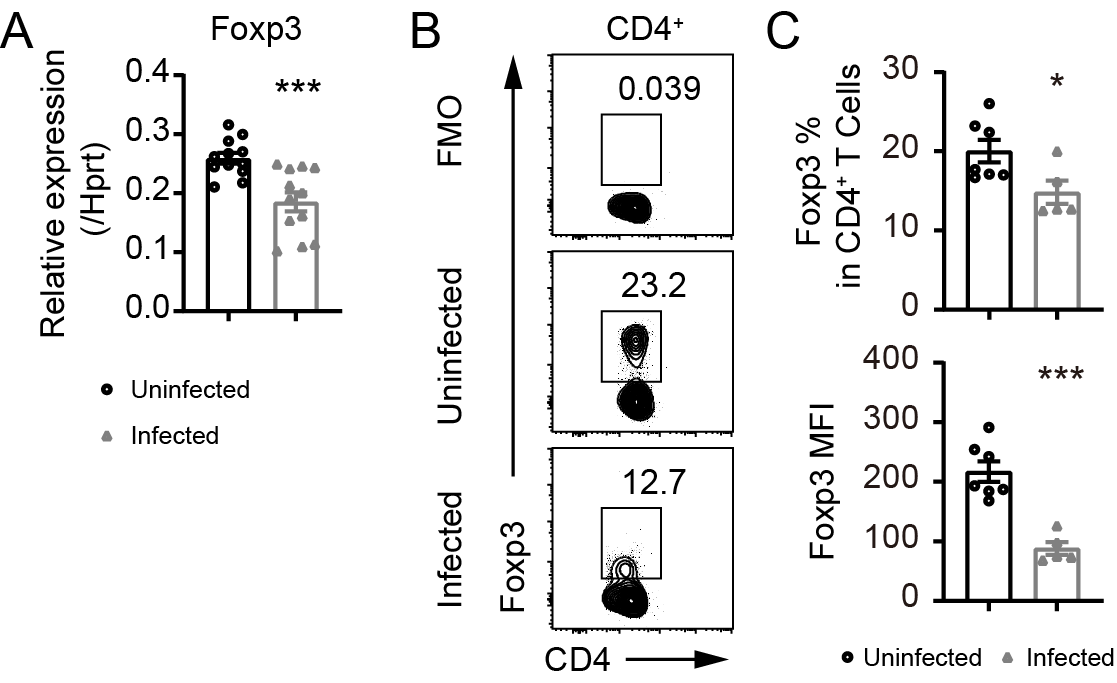

Supplement: S5 Fig — (A) mRNA expression of Foxp3 in T cells of the spleens from WT mice with or without S. japonicum infection. mRNA expression was analyzed with 12 replicates for each group of mice. (B) and (C) FACS analysis of splenic Tregs by Foxp3 staining using WT mice with or without S. japonicum infection (uninfected, n = 7; infected, n = 5). For the infected group, WT mice were infected with S. japonicum for 8 weeks. Data represent the mean ± s.e.m. Statistical significance was assessed by unpaired Student’s t-test or non-parametric unpaired Mann-Whitney test and indicated by * P<0.05, *** P<0.001. (TIF) [file ppat.1010596.s008.tif]

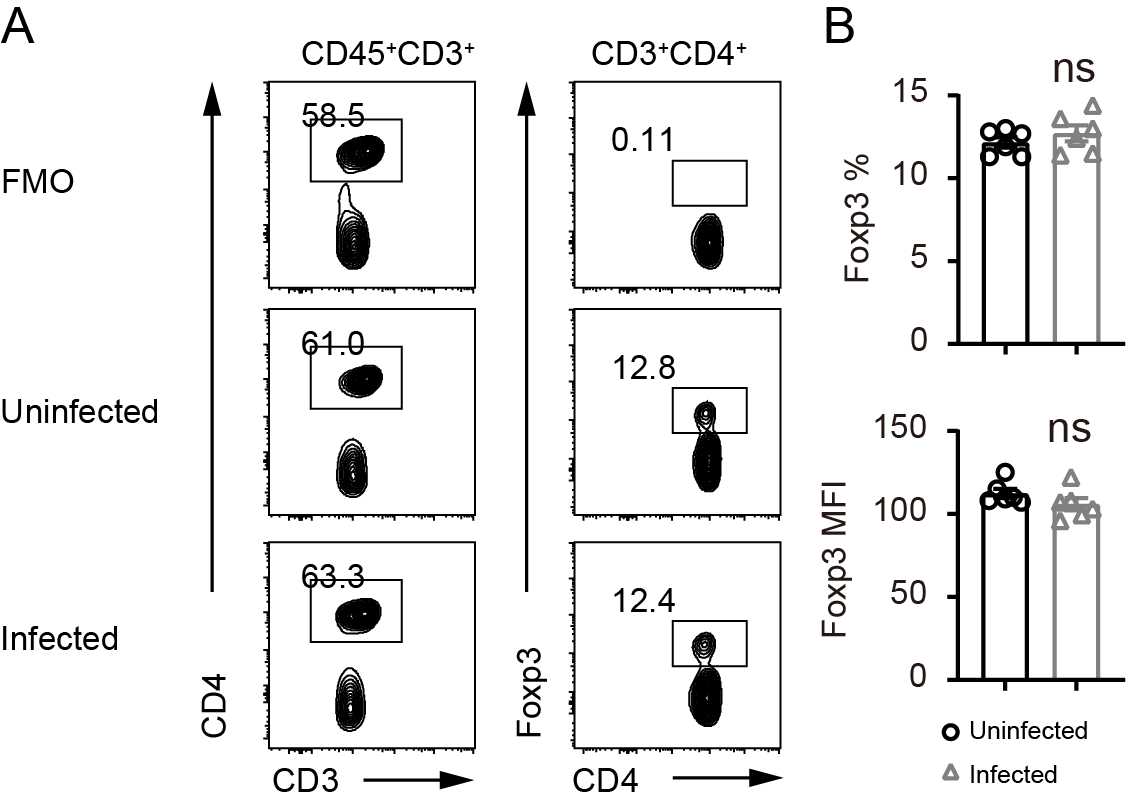

Supplement: S6 Fig — (A-B) Flow cytometric analyses of Foxp3 expression in CD4+ T cells in spleens of C57BL/6 uninfected controls and mice infected with 20 S. japonicum cercariae for 4 weeks (WT mice, n = 6; KO mice, n = 6). Data represent the mean ± s.e.m. Statistical significance was assessed by unpaired Student’s t-test or non-parametric unpaired Mann-Whitney test and indicated by ns, non-significant. (TIF) [file ppat.1010596.s009.tif]

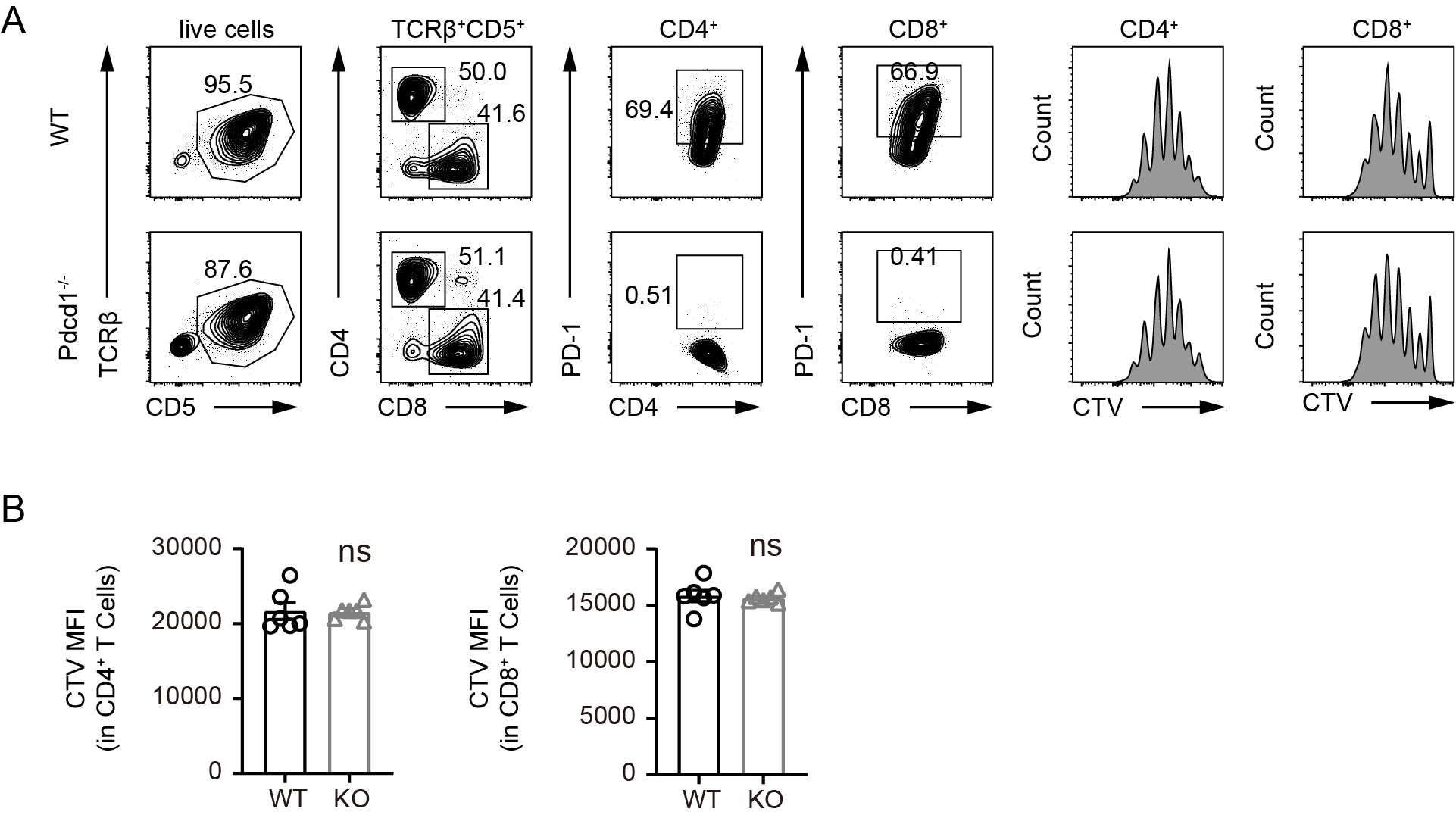

Supplement: S7 Fig — (A-B) Flow cytometric analyses of CTV dilution in CD4+ and CD8+ T cells collected from spleens of WT and PD-1-deficient Pdcd1−/− mice, both of which were infected with S. japonicum for 8 weeks. T cells were stimulated in vitro by treatment with coated anti-CD3 (3 μg/mL) and soluble anti-CD28 (1 μg/mL) antibodies for 72 h. Statistic comparisons were made between two group of mice for MFI of CTV in both CD4+ and CD8+ T cells (WT mice, n = 6; KO mice, n = 6). Data represent the mean ± s.e.m. Statistical significance was assessed by unpaired Student’s t-test or non-parametric unpaired Mann-Whitney test and indicated by ns, non-significant. (TIF) [file ppat.1010596.s010.tif]

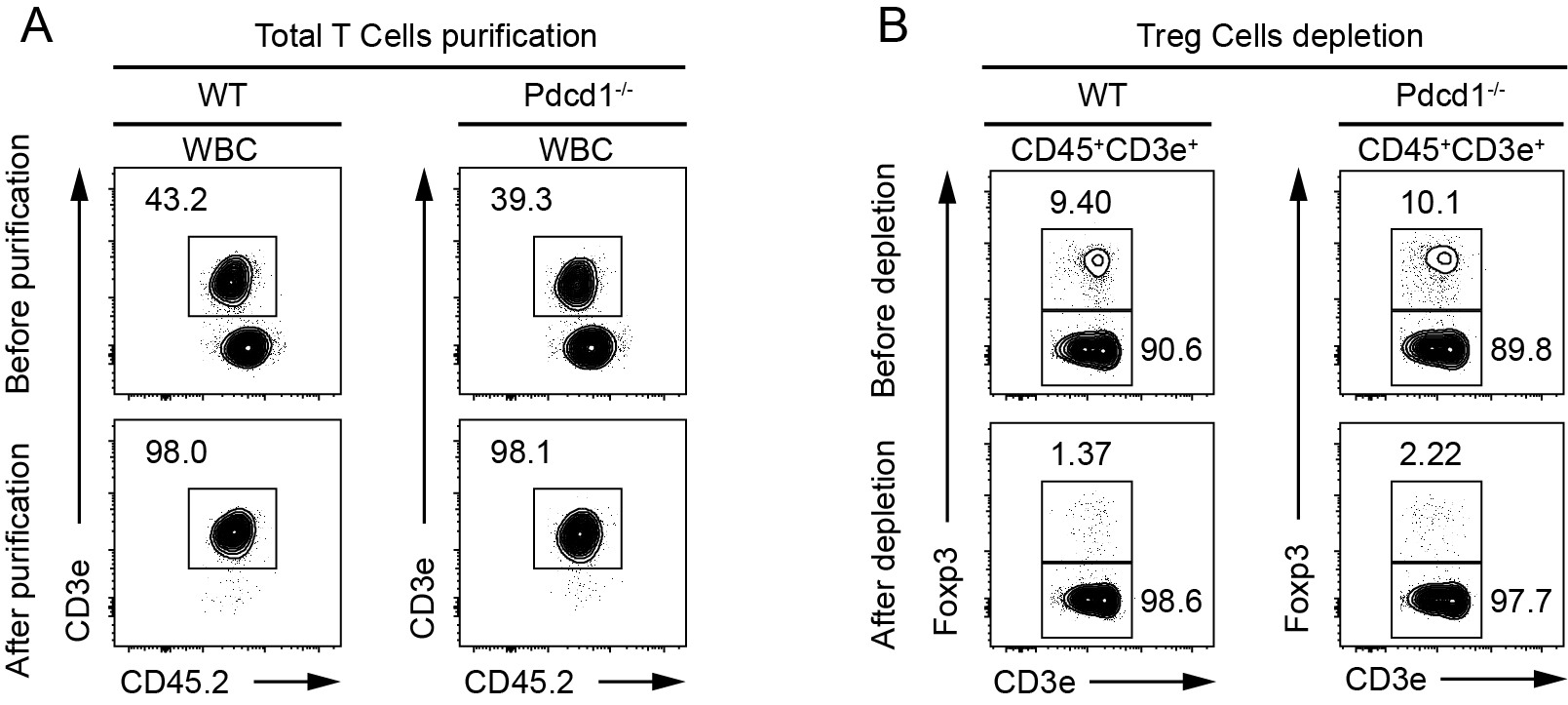

Supplement: S8 Fig — (A) Total cells from lymph nodes from two group of mice were subjected to purification with Dynabeads respectively, purity of T cells from each genotype were verified by FACS. (B) Purified WT and PD-1-deficient total T cells were incubated with anti-CD25 biotinylated antibody followed by washes and incubation with magnetic streptavidin beads for depletion of CD25+ cells. Representative FACS data of Foxp3 intracellular in purified T cells before and after Treg depletion in WT and PD-1-deficient T cells. (TIF) [file ppat.1010596.s011.tif]

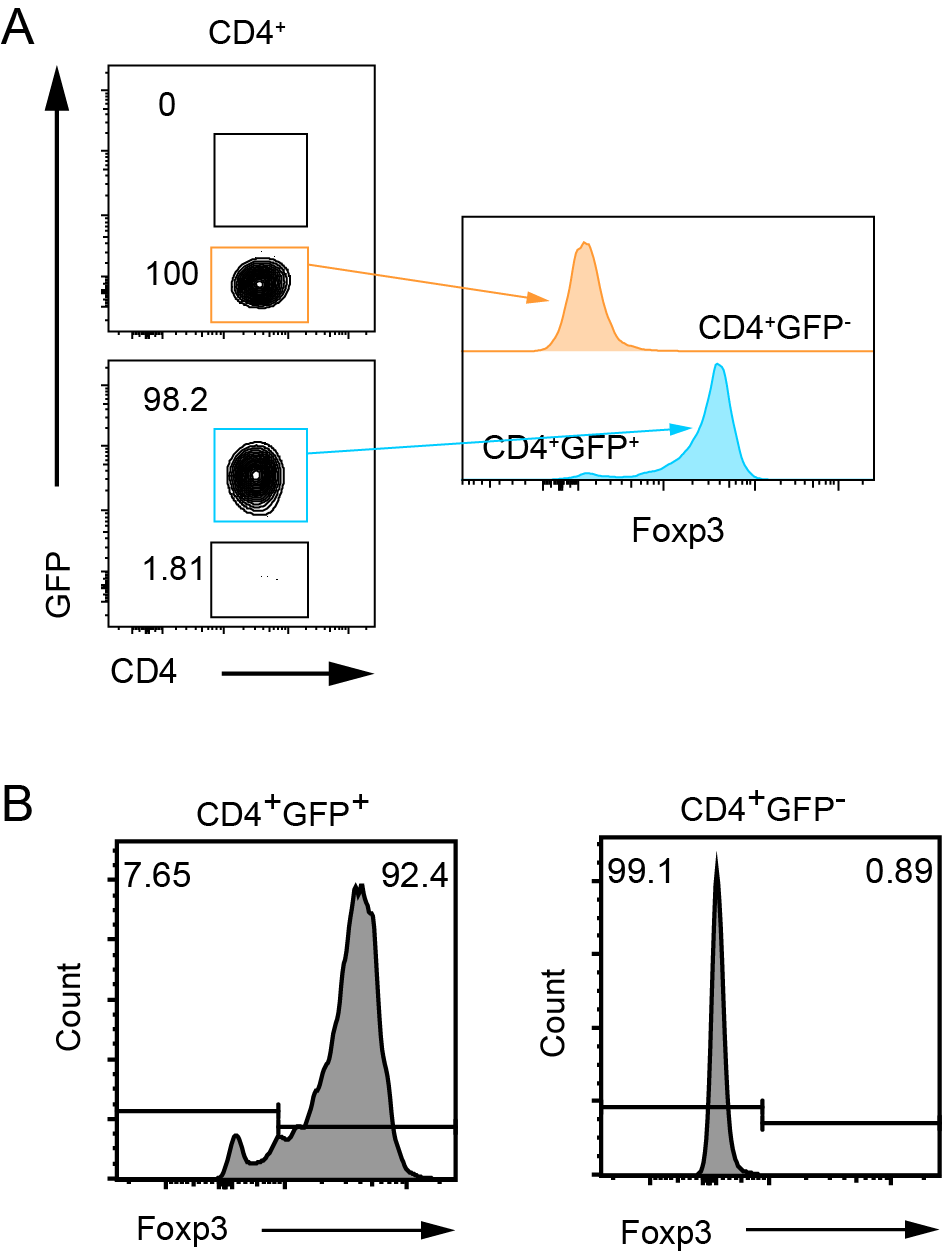

Supplement: S9 Fig — (A-B) Foxp3 staining was tested for sorted Foxp3-eGFP-negative and positive T cells before adoptive transfer. 2 × 10^6^ sorted GFP-negative T cells from CD45.2+ Foxp3-eGFP+ Pdcd1−/− mice and CD45.1+ Foxp3-eGFP+ Pdcd1+/+ respectively were mixed in a 1:1 ratio for each recipient mouse. (TIF) [file ppat.1010596.s012.tif]

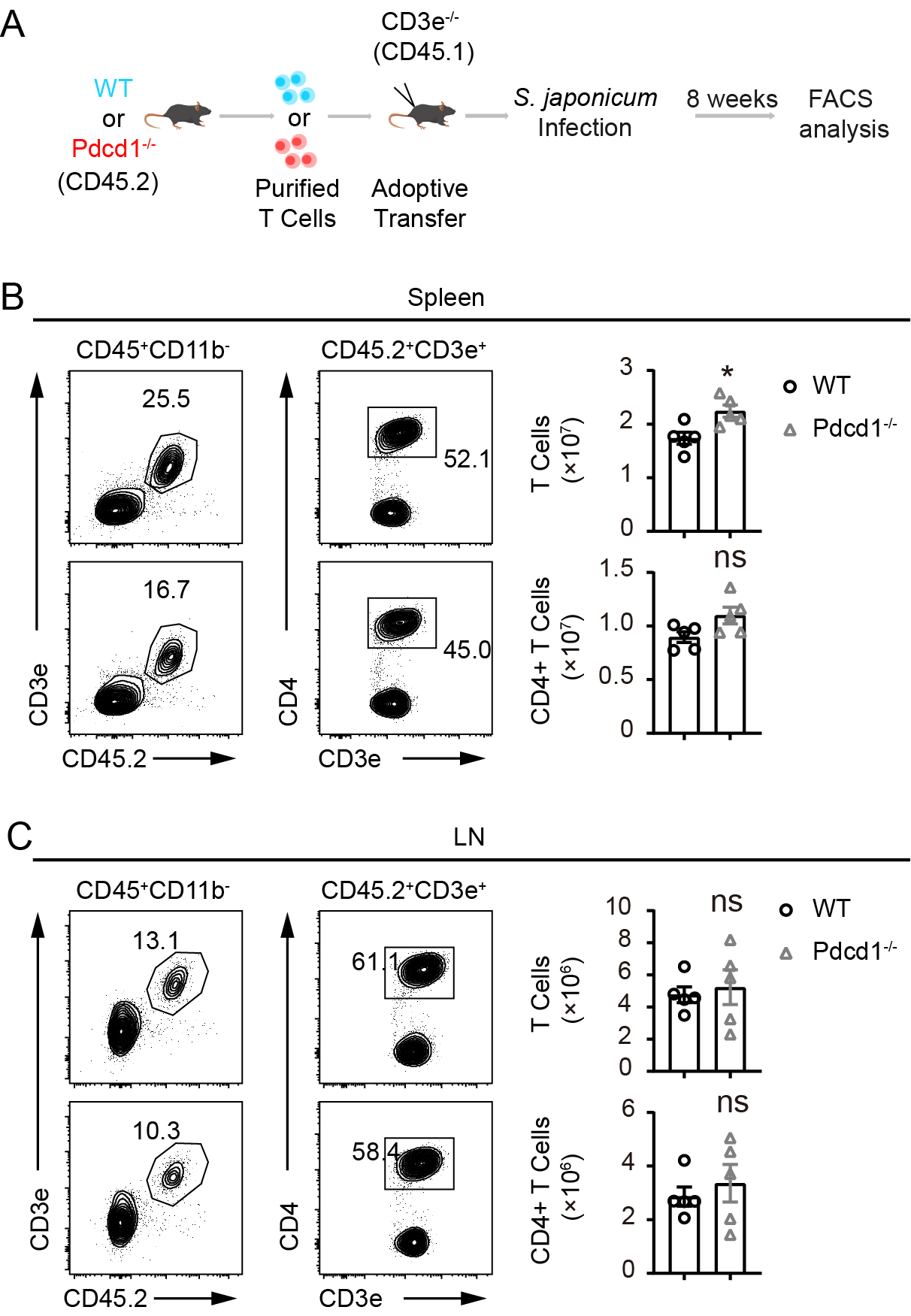

Supplement: S10 Fig — (A) Schematic presentation of the experiments involving total T cell purification, and transfer of equal number of total T cells (with Tregs) into CD45.1+ CD3e−/− mice before S. japonicum infection. (B) Flow cytometric analyses of T cells from WT and PD-1-deficient T cells collected from the spleen of recipient mice 8 weeks after S. japonicum infection. Total T cell counts and CD4+ T cell counts were presented between two group of adoptively transferred mice. (C) Flow cytometric analyses of T cells from WT and PD-1-deficient T cells collected from the livers of recipient mice after S. japonicum infection. Total T cell counts and CD4+ T cell counts were presented between two group of adoptively transferred mice. The two groups of recipient mice (WT group, n = 5; KO group, n = 5) adoptively transferred with purified WT and PD-1-deficient T cells, respectively were infected with S. japonicum for 8 weeks before FACS analyses. 8×10^6^ purified WT and PD-1-deficient T cells (with Tregs) were respectively transferred into each CD45.1+ CD3e−/− recipient mice before S. japonicum infection for 8 weeks. Data represent the mean ± s.e.m. Statistical significance was assessed by unpaired Student’s t-test or non-parametric unpaired Mann-Whitney test and and indicated by * P<0.05, ns, non-significant. (TIF) [file ppat.1010596.s013.tif]

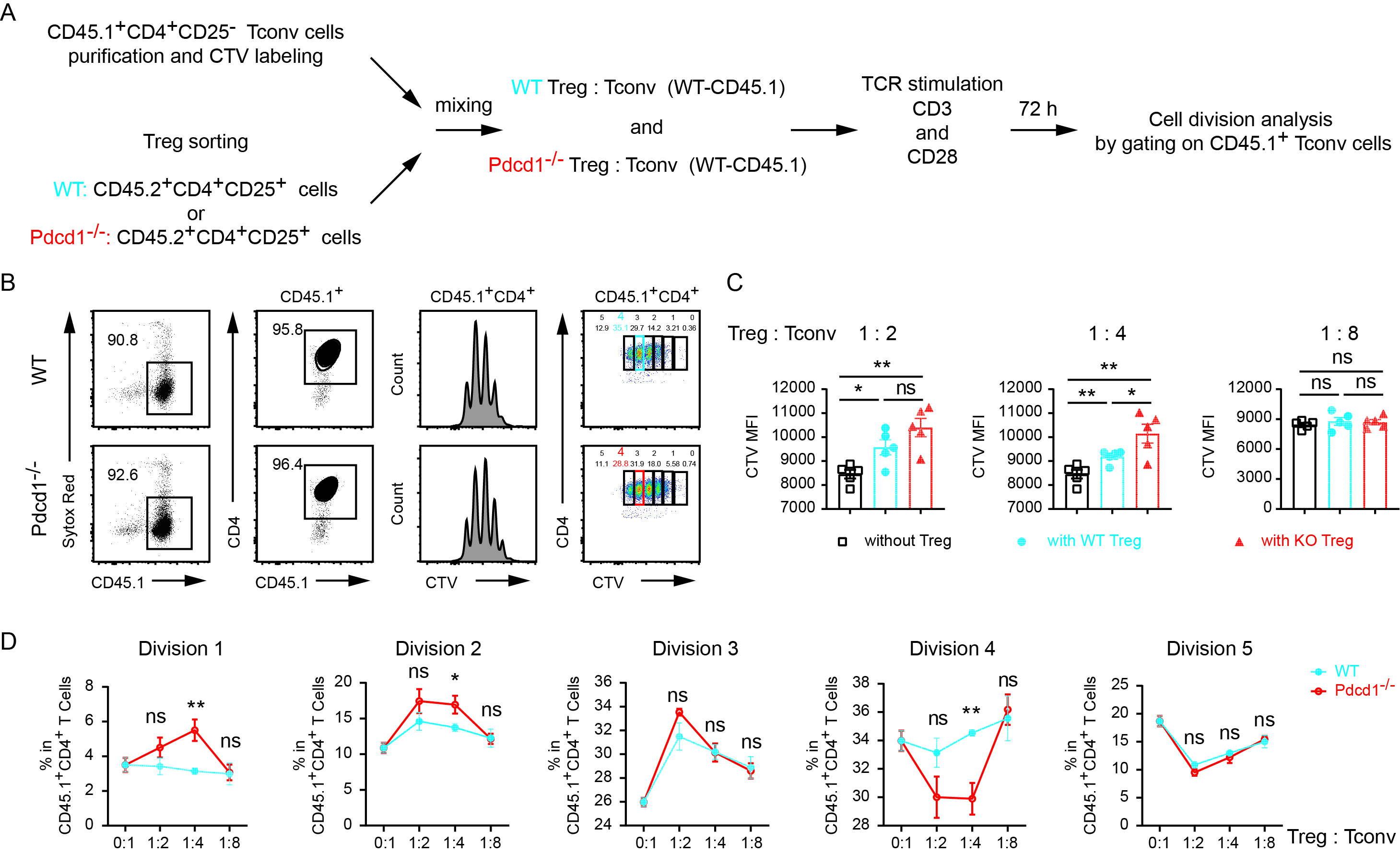

Supplement: S11 Fig — (A) Schematic workflow of the experiment which includes multiple steps such as isolation of conventional CD4+ T cell from CD45.1 congenic mice by untouched mouse CD4 T cell Dynabeads supplemented with rat anti-mouse CD25 monoclonal antibody, cell sorting of two types of Tregs from PD-1 knockout and WT mice, mixture of WT or PD-1-deficient Tregs and CD4 Tconv cells in different ratios, TCR stimulation (anti-CD3 antibody 1 μg/mL and anti-CD28 antibody 1 μg/mL) in vitro and cell division analysis. (B) Representative FACS analyses of T cell divisions by gating CTV labelled CD45.1+ CD4 Tconv with TCR stimulation for 72 h, in the presence of WT or PD-1-deficient Tregs at the Treg:Tconv ratio of 1:4. (C) Statistic comparisons of CTV fluorescence attenuation between CD45.1+ CD4 Tconv cells, which were co-cultured with WT or PD-1-deficient Tregs at the Treg:Tconv ratios of 0:1, 1:2, 1:4 and 1:8. Empty squares represent data points of CTV levels from CD45.1+ CD4 Tconv cells without co-culture of Tregs. Solid blue dots represent data points of CTV levels from CD45.1+ CD4 Tconv cells co-cultured with WT Tregs, and red triangles represent data points of CTV levels from CD45.1+ CD4 Tconv cells co-cultured with PD-1 knockout Tregs. (D) Percentage of cell divisions analyzed by gating CD45.1+ CD4 Tconv cells with TCR stimulation for 72 h. Division 4 and 5 indicate cells undergoing more divisions and faster cell expansion, and Division 1 and 2 indicate cells with less divisions and slower cell expansion. Among two group of CD45.1+ CD4 Tconv cells co-cultured with either WT Tregs or PD-1 knockout Tregs, lower percentage of fast-growing cells indicates stronger Treg-mediated suppression. n = 5 repeats for all the conditions. Data represent the mean ± s.e.m. Statistical significance was assessed by unpaired Student’s t-test or non-parametric unpaired Mann-Whitney test and indicated by * P<0.05, ** P<0.01, ns, non-significant. (TIF) [file ppat.1010596.s014.tif]

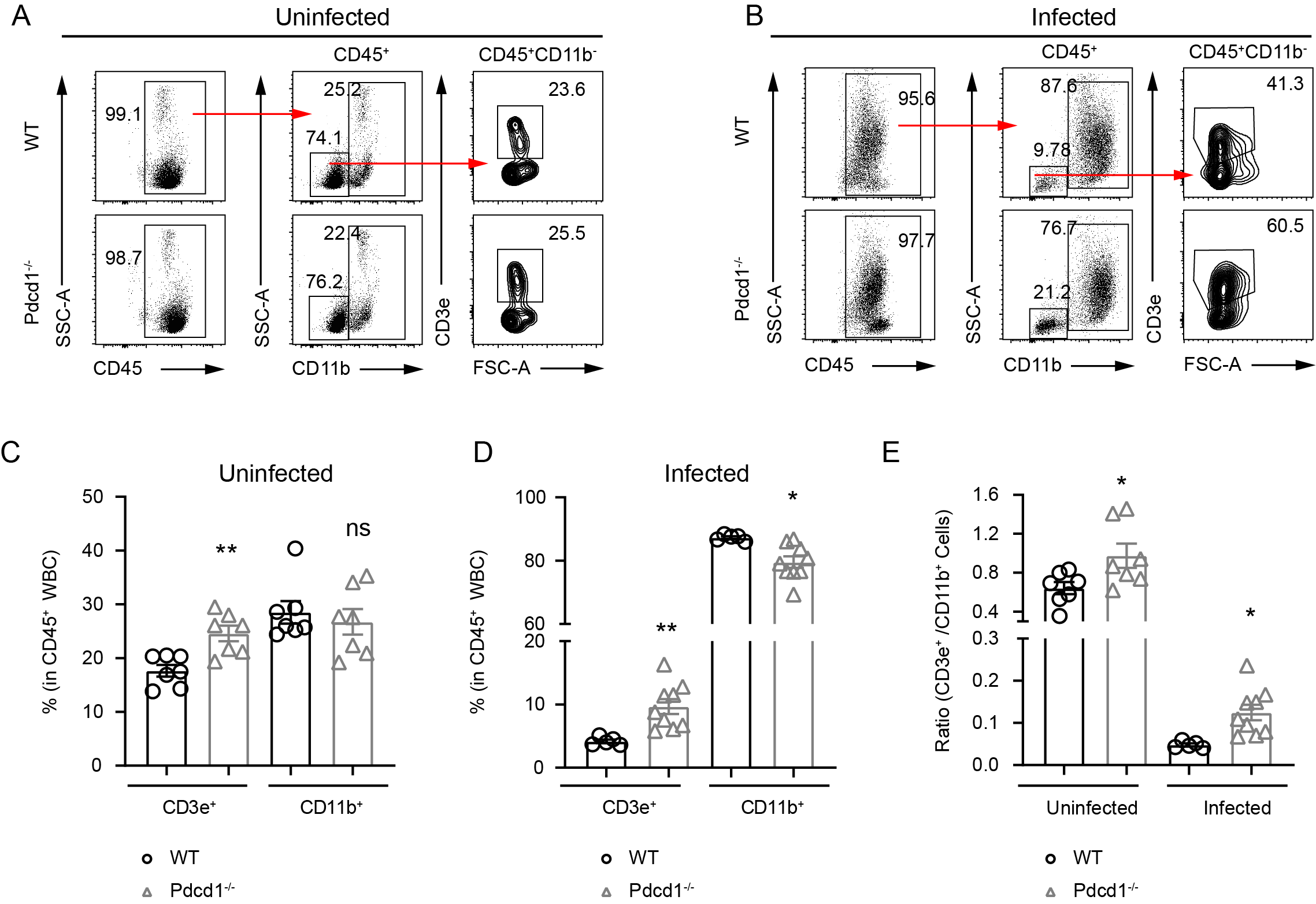

Supplement: S12 Fig — (A) In livers of un-infected mice, FACS gating for frequencies of CD11b+ myeloid cells and CD3+ T cells involving both WT and PD-1-deficient animals. (B) In livers of S. japonicum infected mice, FACS gating for frequencies of CD11b+ myeloid cells and CD3+ T cells involving both WT and PD-1-deficient animals (WT, n = 7; KO, n = 7). (C) In livers of un-infected mice, statistic comparisons for frequencies of CD11b+ myeloid cells and CD3+ T cells involving both WT and PD-1-deficient animals. (D) In livers of S. japonicum infected mice, statistic comparisons for frequencies of CD11b+ myeloid cells and CD3+ T cells involving both WT and PD-1-deficient animals (WT, n = 5; KO, n = 9). (E) Ratio of CD3+ cells to CD11b+ cells in the infected and un-infected animals, the same data points from (C) and (D) were used for calculation of the ratio. Data represent the mean ± s.e.m. Statistical significance was assessed by unpaired Student’s t-test or non-parametric unpaired Mann-Whitney test and indicated by * P<0.05, ** P<0.01, ns, non-significant. (TIF) [file ppat.1010596.s015.tif]

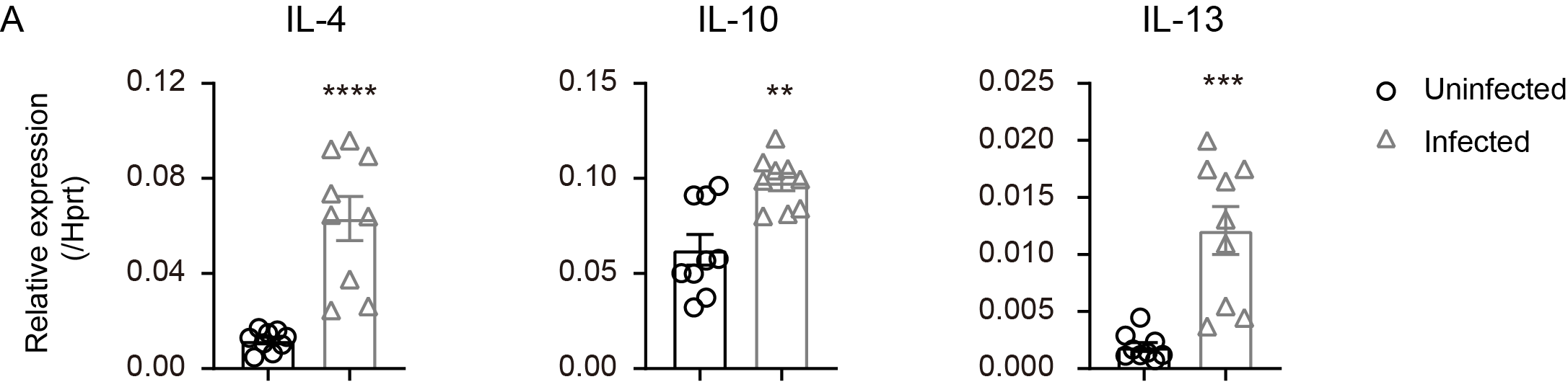

Supplement: S13 Fig — For the infected group, WT mice were infected with S. japonicum for 8 weeks. Each sample was analyzed with 9 replicates. Data represent the mean ± s.e.m. Statistical significance was assessed by unpaired Student’s t-test or non-parametric unpaired Mann-Whitney test and indicated by ** P<0.01, *** P<0.001, **** P<0.0001. (TIF) [file ppat.1010596.s016.tif]
